# Sepsis reporting signals associated with endothelin receptor antagonists and IFITM3-Centered interferon-responsive monocyte features: a pharmacovigilance and transcriptomic study

**DOI:** 10.3389/fphar.2026.1864683

**Published:** 2026-07-16

**Authors:** Zekun Cheng, Jiahang Li, Feng Liu

**Affiliations:** 1 National Clinical Research Center for Geriatric Disorders, Xiangya Hospital, Central South University, Changsha, Hunan, China; 2 Xiangya School of Medicine, Central South University, Changsha, Hunan, China; 3 Department of Oncology, Xiangya Hospital, Central South University, Changsha, Hunan, China; 4 Third Hospital of Changsha, Changsha, Hunan, China

**Keywords:** endothelin receptor antagonists, IFITM3, interferon signaling, machine learning, molecular dynamics simulation, monocyte dysregulation, pulmonary arterial hypertension, riociguat

## Abstract

**Background:**

Whether pulmonary arterial hypertension (PAH)–targeted therapies, particularly endothelin receptor antagonists (ERAs), are associated with disproportionate sepsis reporting in real-world pharmacovigilance data remains insufficiently explored. The monocyte transcriptional states that characterize sepsis-related immune dysregulation and may provide biological context for such reporting signals are also incompletely defined.

**Methods:**

We constructed an integrated, hypothesis-generating analytical framework incorporating: (i) FDA Adverse Event Reporting System (FAERS) disproportionality analysis coupled with XGBoost-based modeling for pharmacovigilance signal detection; (ii) single-cell RNA sequencing (scRNA-seq) analysis of peripheral blood mononuclear cells with intercellular communication inference; (iii) weighted gene co-expression network analysis (WGCNA) and cytoHubba-based topological prioritization, combined with an ensemble machine learning framework for diagnostic signature construction; and (iv) molecular docking and 100-nanosecond all-atom molecular dynamics (MD) simulation.

**Results:**

FAERS analysis identified Maitentan and ambrisentan as PAH-targeted therapies with positive reporting signals for the MedDRA Preferred Term “Sepsis,” with adjusted reporting associations persisting after adjustment for available demographic variables. Sex-stratified analysis showed marked heterogeneity in reporting signals, although these findings may be influenced by the sex distribution of PAH populations and other unmeasured confounders. ScRNA-seq resolved seven monocyte subpopulations, among which the interferon-responsive Mono_IFN subset—marked by IFIT1, ISG15, and IFITM3 expression—occupied a signaling hub position within the IFN-γ communication network and expanded in sepsis-associated states. Systematic comparison of 112 integrated machine learning algorithm combinations based on cytoHubba-prioritized genes yielded a 29-gene diagnostic model with cross-cohort discrimination for sepsis. Transcriptional co-expression analysis nominated IFITM3, a marker of the interferon-responsive monocyte state, as a candidate node connecting the diagnostic signature with interferon-related immune dysregulation. Molecular docking and 100-nanosecond MD simulation suggested a structurally stable riociguat–IFITM3 interaction *in silico*. This finding remains exploratory and requires biochemical and functional validation.

**Conclusion:**

This integrated pharmacovigilance and transcriptomic study identifies sepsis-reporting signals associated with selected endothelin receptor antagonists and characterizes an IFITM3-associated interferon-responsive monocyte state in sepsis datasets. These findings should be interpreted as reporting associations and transcriptomic hypotheses rather than evidence of causal drug-induced sepsis. The predicted riociguat–IFITM3 interaction provides a computational hypothesis for future experimental validation.

## 1 Introduction

Sepsis is defined as a life-threatening organ dysfunction caused by a dysregulated host response to infection (Singer et al., 2016). According to estimates from the Global Burden of Disease (GBD) study, approximately 48.9 million cases of sepsis and 11.0 million sepsis-related deaths were recorded worldwide in 2017, accounting for 19.7% of all global deaths (Rudd et al., 2020). More recent analyses from the updated GBD 2021 study further highlight a concerning trend: between 2019 and 2021, sepsis-related mortality attributable to infectious etiologies increased by 86.4%, reaching 15.5 million deaths, thereby reversing the declining trajectory observed over the preceding decades (GBD 2021 Global Sepsis Collaborators, 2025).

The pathogenesis of sepsis fundamentally arises from a global collapse of immune homeostasis, in which the host innate immune response—particularly that mediated by myeloid cells—shifts from protective antimicrobial defense to a self-destructive inflammatory cascade (Cao et al., 2023). Monocytes and macrophages occupy a central hub in this process. During the hyperinflammatory phase, they serve as major sources of pro-inflammatory cytokines, including TNF-α, IL-1β, and IL-6, thereby directly driving the cytokine storm that contributes to endothelial barrier disruption and multi-organ failure (Takahama et al., 2024; Maneta et al., 2023).

Advances in single-cell RNA sequencing (scRNA-seq) have further delineated the cellular heterogeneity underlying this dysregulation. Notably, sepsis induces the expansion of a distinct CD14^+^ monocyte state, termed MS1, characterized by suppressed HLA-DR expression and elevated levels of RETN, ALOX5AP, and IL1R2. Functionally, this population exhibits pronounced immunosuppressive activity and is strongly associated with disease severity and mortality (Reyes et al., 2020). Complementary multi-cohort consortium analyses have proposed that myeloid and lymphoid dysfunction constitute two orthogonal axes of immune perturbation, each independently correlating with adverse outcomes in sepsis and other critical illnesses such as acute respiratory distress syndrome (ARDS) (Moore et al., 2025).

Despite these mechanistic insights, robust biomarkers capable of capturing early, preclinical immune dysregulation—particularly at the resolution of monocyte subset heterogeneity—remain lacking in clinical practice (Evans et al., 2021).

Sepsis-associated immune dysregulation is increasingly recognized as a heterogeneous and state-dependent process rather than a uniform hyperinflammatory syndrome. In different patients, or even at different stages within the same patient, excessive innate immune activation may coexist with impaired antigen presentation, lymphocyte dysfunction, and features of immunoparalysis. This complexity is clinically relevant because immune-modulating therapies may exert divergent effects depending on the host immune state. For example, interferon-γ has been reported to restore monocyte HLA-DR expression in selected patients with sepsis-associated immunoparalysis, whereas interleukin-1 receptor blockade showed a potential survival benefit mainly in a subgroup of sepsis patients with hepatobiliary dysfunction and disseminated intravascular coagulation. These examples suggest that drug–immune interactions in sepsis are highly context dependent and may be missed when patients are analyzed only at the syndrome level.

Beyond classical immunosuppressive or anti-inflammatory agents, drugs developed for non-immune indications may also affect host inflammatory responses by modulating vascular–immune crosstalk, endothelial activation, cytokine networks, or myeloid-cell polarization. The endothelin pathway is particularly relevant in this regard. Endothelin-1 is not only a potent vasoactive mediator but has also been implicated in inflammatory signaling, endothelial–immune interactions, and sepsis-related vascular dysfunction. Therefore, evaluating whether endothelin pathway–targeting drugs are associated with infection-related adverse-event signals may provide a useful pharmacovigilance entry point for exploring underrecognized drug-related immune perturbations in sepsis (Dastan et al., 2024; Choubisa et al., 2025; Ra and mos, 2025).Pulmonary arterial hypertension (PAH) is a progressive vascular disorder characterized by endothelial dysfunction, smooth muscle cell proliferation, and a sustained increase in pulmonary vascular resistance**.** Endothelin receptor antagonists (ERAs), including the dual ETA/ETB antagonist Maitentan and the selective ETA antagonist ambrisentan, constitute a cornerstone of targeted pharmacotherapy for PAH (Pulido et al., 2013; Peacock et al., 2015).

Beyond its well-established role as a potent vasoconstrictor, endothelin-1 (ET-1) exerts broad immunomodulatory effects. It can activate monocytes and neutrophils, induce pro-inflammatory cytokine production via NF-κB signaling, and promote macrophage polarization toward an M1-like phenotype (Kowalczyk et al., 2015). Overexpression of ET-1 has also been shown to upregulate endothelial ICAM-1 and monocyte chemoattractant protein-1 (MCP-1), thereby facilitating macrophage recruitment and inflammatory mediator release (Banecki and Dora, 2023).

Given the widespread expression of endothelin receptors on T and B lymphocytes as well as myeloid cells, ERA-mediated receptor blockade may substantially reshape IFN-γ production and the activation landscape of immune cells (Elisa et al., 2015). However, whether chronic ERA exposure disrupts systemic immune homeostasis and consequently elevates the risk of sepsis remains largely unexplored, both at the epidemiological and mechanistic levels.

To address this gap, we established a multi-layered, hypothesis-generating framework integrating pharmacovigilance signal detection with transcriptomic and structural analyses. First, we performed large-scale disproportionality analyses using FAERS data and applied XGBoost-based modeling to identify PAH-targeted therapies associated with disproportionate sepsis reporting. These analyses were designed for safety-signal detection rather than causal risk estimation. We then analyzed public single-cell RNA sequencing datasets derived from sepsis patients and controls to characterize sepsis-associated monocyte states, with particular attention to interferon-responsive monocyte programs. Importantly, these datasets did not include ERA-exposed PAH patients who developed sepsis; therefore, the identified Mono_IFN population should be interpreted as a sepsis-associated interferon-responsive monocyte state rather than a drug-induced mediator of ERA-associated sepsis reporting. Furthermore, WGCNA, cytoHubba-based topological prioritization, and ensemble machine learning were used to construct a diagnostic transcriptomic signature and to identify candidate immune-network genes associated with sepsis-related dysregulation. Within this framework, IFITM3 emerged as an interferon-responsive marker and network-associated node rather than a validated causal mediator of drug-associated sepsis. Finally, molecular docking and all-atom molecular dynamics simulations were performed to explore a computationally predicted riociguat–IFITM3 interaction. Collectively, this study aims to generate pharmacovigilance and transcriptomic hypotheses by separately characterizing ERA-associated sepsis reporting signals, sepsis-associated interferon-responsive monocyte states, diagnostic immune signatures, and exploratory structural predictions, while recognizing that controlled clinical and experimental studies are required to establish causality or therapeutic relevance.

## 2 Methods

### 2.1 Data acquisition and preprocessing

Real-world pharmacovigilance data were obtained from the U.S. Food and Drug Administration (FDA) Adverse Event Reporting System (FAERS), covering the period from the first quarter of 2014 to the fourth quarter of 2024. Data cleaning and standardization were conducted following a stringent and reproducible pipeline.

First, duplicate reports were removed based on PRIMARYID and CASEID to minimize redundancy-induced bias. Patient demographic variables (e.g., sex and age) and adverse event–related fields were subsequently harmonized through standardized coding. Within each case, duplicate drug entries were eliminated, retaining only unique CASEID–DRUGNAME pairs to ensure data integrity. To enhance causal relevance, only records with drug role codes (ROLE_COD) explicitly annotated as Primary Suspect (PS) or Secondary Suspect (SS) were included in downstream analyses.

Adverse events were standardized using MedDRA version 25.0. The primary endpoint was defined as reports containing the Preferred Term “Sepsis.” Other related MedDRA terms, including “Severe sepsis,” “Septic shock,” “Bacterial sepsis,” and infection-site-specific sepsis terms, were not included in the primary endpoint definition. Therefore, the FAERS analysis was restricted to sepsis reporting based on the PT “Sepsis.”

To mitigate confounding introduced by supportive care interventions, reports in which non-specific agents (e.g., electrolyte infusion solutions) were designated as primary suspect drugs were excluded. For continuous variables such as age and body weight, extreme outliers exceeding physiological plausibility (e.g., adult body weight >300 kg or <1 kg; age >120 years) were recoded as missing values (NA). In global epidemiological analyses, records with missing values were retained and categorized as “unknown” to maximize sample utilization, whereas in stratified analyses, records lacking the corresponding variables were excluded (Supplementary Figure S1).

Continuous variables, including age and time-to-onset, were summarized using the median and interquartile range (IQR). All data preprocessing and statistical analyses were conducted in R (version 4.4.2).

Single-cell RNA-seq datasets of peripheral blood mononuclear cells from patients with sepsis and controls were retrieved from the Gene Expression Omnibus (GEO) database under accession numbers GSE279452. Bulk transcriptomic datasets were obtained from GEO, including GSE65682 as the training cohort and GSE95233, GSE13904, GSE185263, GSE69528, and GSE63042 as external validation cohorts. The source database, accession number, platform, sample type, disease/control definition, sample size, and analytical role of each dataset are summarized in Supplementary Table S1.

### 2.2 Clinical endpoint definition and disproportionality analysis

Adverse events were standardized using the Medical Dictionary for Regulatory Activities (MedDRA®, version 25.0). The primary clinical endpoint was defined as reports containing the Preferred Term (PT) “Sepsis.” This PT was selected as the sole endpoint to maintain a focused and reproducible phenotype for pharmacovigilance signal detection. Other MedDRA terms, including “Severe sepsis,” “Septic shock,” “Bacterial sepsis,” and infection-site-specific sepsis terms, were not included in the primary endpoint definition. Therefore, throughout the manuscript, the endpoint is referred to as sepsis reporting or reports with the PT “Sepsis,” rather than severe sepsis.

Potential drug-event reporting associations were quantified using a classical 2 × 2 contingency table framework. In this matrix, a denotes the number of reports in which the target drug and the PT “Sepsis” co-occurred; b represents reports in which the target drug was reported with non-target adverse events; c represents reports in which non-target drugs were reported with the PT “Sepsis”; and d denotes reports in which non-target drugs were reported with non-target adverse events.

To mitigate spurious associations driven by rare events, multiple complementary disproportionality metrics were computed, including the reporting odds ratio (ROR), proportional reporting ratio (PRR), and the Bayesian shrinkage–based information component (IC). A positive pharmacovigilance signal was defined using a composite criterion: (i) the number of co-occurrence reports (a) ≥ 5; (ii) the lower bound of the one-sided 95% confidence interval for ROR (ROR025) > 1.0; and (iii) the lower bound of the one-sided 95% confidence interval for IC (IC025) > 0.

To further characterize the temporal distribution of sepsis reports associated with target drugs, time-to-onset (TTO) distributions were modeled using Weibull distribution fitting. The shape parameter (β) was used to describe reporting-time patterns, with β < 1 indicating an early-onset reporting profile rather than a causal hazard pattern.

Multivariable logistic regression was used to evaluate whether drug-event reporting associations persisted after adjustment for available demographic variables, including age group, sex, and body-weight group where applicable. Missing demographic values were treated as an “unknown” category in global models to preserve sample size. Because FAERS does not systematically capture PAH severity, hospitalization status, central venous access, infection history, comorbidities, concomitant prostacyclin therapy, immunosuppressive exposure, or detailed polypharmacy, these models were not intended to estimate causal effects or establish independent clinical risk factors. Adjusted estimates were therefore interpreted as adjusted reporting associations.

### 2.3 scRNA-seq quality control, batch correction, and preprocessing

Raw count matrices from single-cell RNA sequencing (scRNA-seq) datasets were processed using the Seurat package (version 5.2.1) in R. To ensure the inclusion of high-quality cells and minimize technical artifacts, stringent quality-control criteria were applied before downstream analysis. Cells were retained if they met the following thresholds: 300 < nFeature_RNA < 7,000, mitochondrial transcript proportion (percent.mt) < 20%, and hemoglobin gene proportion (percent.hb) < 1%. Cells outside these thresholds were excluded from further analysis.

To reduce the influence of potential doublets generated by droplet-based sequencing, doublet detection was performed using DoubletFinder. The expected doublet formation rate was set to 25%, and cells predicted as doublets were removed before normalization and dimensionality reduction.

The filtered expression matrix was normalized using the LogNormalize method with a scale factor of 10,000. The top 2,000 highly variable genes (HVGs) were identified using the FindVariableFeatures function with the “vst” method. The normalized data were then centered and scaled using ScaleData. Principal component analysis (PCA) was performed based on the selected HVGs. To mitigate batch effects across samples and improve cross-sample comparability, Harmony was applied in PCA space for batch correction. The Harmony-corrected embeddings were subsequently used for nearest-neighbor graph construction, clustering, and UMAP visualization.

### 2.4 Dimensionality reduction, clustering, and monocyte subpopulation annotation

Cellular subpopulations were identified using a two-step unsupervised clustering strategy. First, a global immune cell atlas was constructed to identify major immune cell lineages. Based on dimensionality assessment and clustering stability, the top 20 Harmony-corrected principal components were used to construct a shared nearest-neighbor graph. Clustering was performed using the Louvain algorithm implemented in the FindClusters function, with the resolution parameter set to 0.3. Uniform Manifold Approximation and Projection (UMAP) was then applied for two-dimensional visualization. Major immune cell types, including T cells, B cells, NK cells, plasma cells, and myeloid cells, were annotated according to canonical marker genes.

To further resolve heterogeneity within the myeloid compartment, myeloid cells were subsetted from the global atlas and reprocessed independently. Highly variable genes were recalculated, PCA was repeated, and Harmony-corrected principal components were used for refined clustering. For myeloid subclustering, the resolution parameter was set to 0.5 to capture finer monocyte and dendritic-cell states. UMAP visualization was performed based on the refined myeloid-cell embedding.

Differentially expressed genes for each refined myeloid cluster were identified using the FindAllMarkers function with the Wilcoxon rank-sum test. Genes with adjusted P < 0.05 and |log_2_ fold change| > 0.25 were considered significant marker genes. Monocyte and myeloid subpopulations were annotated based on cluster-specific marker genes and functional transcriptional programs. Specifically, interferon-responsive monocytes were defined by elevated expression of interferon-stimulated genes, including IFIT1, IFITM3, ISG15, and OAS1; inflammatory monocytes were characterized by pro-inflammatory markers such as S100A8, S100A9, IL1B, and LST1; resident-like monocytes were annotated according to genes such as LYZ, CTSD, CFD, and MAFB; stress-related monocytes were identified by stress-response genes including JUN, FOS, MALAT1, and NEAT1; and conventional dendritic cells were annotated based on markers such as CD1C, FCER1A, and HLA-DQA1.

To support functional annotation at single-cell resolution, pathway activity scores were calculated using AUCell and complementary gene-set scoring methods where appropriate. These analyses were used to evaluate interferon-response activity and other immune programs across the refined myeloid subpopulations, thereby supporting the identification of the Mono_IFN subset and related monocyte states.

### 2.5 Trajectory inference of monocyte polarization using CytoTRACE and Monocle3

To reconstruct the dynamic trajectory of monocyte polarization toward inflammatory states, an integrative framework combining CytoTRACE and Monocle3 was employed. First, CytoTRACE was applied to infer differentiation potential in an unsupervised manner based on gene count signatures derived from single-cell transcriptomes. Cells with the highest CytoTRACE scores were defined as residing at the earliest, least differentiated (progenitor-like) states along the polarization continuum.

Subsequently, the Seurat object was converted into a Monocle3-compatible *cell_data_set* object for trajectory analysis. The principal graph representing pseudotemporal progression was learned in the UMAP embedding using the *learn_graph* function. During trajectory ordering (*order_cells*), the root node was explicitly anchored to regions enriched for high CytoTRACE-scoring cells, thereby enabling biologically informed initialization of pseudotime inference. Pseudotime values were then assigned to all monocytes along the inferred trajectory.

To identify transcriptional programs driving monocyte polarization, spatial autocorrelation analysis was performed using Moran’s I statistic (*graph_test* function) to detect genes exhibiting significant dynamic changes along the trajectory. These genes were further clustered based on their temporal expression patterns using dynamic time warping (DTW), followed by Gene Ontology (GO) enrichment analysis to characterize associated biological processes.

### 2.6 Microenvironmental cell–cell communication and ligand–receptor interaction analysis

To delineate the intercellular communication landscape between the polarized Mono_IFN subpopulation and other immune cells within the microenvironment, the CellChat framework (version 2.1.2) was employed. Leveraging its curated human ligand–receptor interaction database (CellChatDB), overexpressed ligands and receptors in each cell subset were first identified using the *identifyOverExpressedGenes* and *identifyOverExpressedInteractions* functions.

Subsequently, intercellular communication probabilities were inferred based on the law of mass action, integrating ligand–receptor expression levels with the relative abundance of interacting cell populations. This enabled quantitative modeling of signaling pathway activities between specific cell types.

To characterize the topological role of Mono_IFN within the global communication network, network centrality metrics—including information centrality, indegree, and outdegree—were computed. These analyses allowed for systematic evaluation of the extent to which Mono_IFN acts as a signaling hub within pro-inflammatory interaction networks, with particular emphasis on the IFN-γ signaling axis (IFNG and its receptors IFNGR1/IFNGR2).

### 2.7 Transcriptomic preprocessing and feature extraction

Bulk transcriptomic datasets (RNA-seq and microarray) retrieved from the Gene Expression Omnibus (GEO) were processed using a standardized and reproducible pipeline. For microarray datasets, probe-level data were annotated and mapped to gene symbols using platform-specific annotation packages. When multiple probes corresponded to the same gene, the probe with the highest mean expression across samples was retained as the representative.

To enhance biological interpretability, genes were filtered using the *org. Hs.eg.db* database, retaining only protein-coding genes and excluding all non-coding transcripts. To further reduce low-expression noise and enrich for biologically informative features, the median absolute deviation (MAD) was calculated for each gene across samples, and the top 5,000 most variable genes were selected to construct a high-quality expression matrix.

Because the bulk transcriptomic cohorts were profiled on heterogeneous platforms (several microarray platforms and RNA-sequencing), a harmonization procedure was applied to enable cross-platform model development and validation. Analyses were restricted to the consensus genes detected across all six cohorts, defining a common feature space of 33 genes (Supplementary Figure S3D). Within this feature space, each cohort was standardized independently by within-cohort Z-score standardization to remove cohort-specific location and scale differences, and the standardized values were then converted to within-sample ranks (rank transformation), yielding per-sample profiles that are largely independent of platform-specific intensity scales. The cohorts were not pooled, and no cross-cohort batch-correction method (e.g., ComBat) was applied; the diagnostic model was trained on a single cohort (GSE65682) and evaluated on the remaining cohorts without retraining. Because normalization was performed separately within each cohort, no information was shared between the training and validation data during harmonization, and cross-cohort performance was therefore assessed under fully independent normalization.

The adequacy of this procedure was evaluated using batch-effect diagnostics (Supplementary Figure S3). The 33 consensus genes were complete across all cohorts (Supplementary Figure S3D), and principal-component and UMAP analyses showed substantial overlap among the microarray cohorts (Supplementary Figure S3E–G). GSE63042 separated partially along the first principal component, consistent with its distinct platform (RNA-sequencing) and control composition (SIRS rather than healthy individuals) rather than with systematic technical artifact.

### 2.8 Trait quantification and weighted gene Co-expression network analysis (WGCNA)

To establish a link between bulk transcriptomic profiles and single-cell–derived cellular states, gene signatures representing Mono_IFN and Mono_active subpopulations were derived from single-cell analysis by selecting the top 50 marker genes for each subset. In addition, a curated set of 24 genes encoding the molecular targets of the PAH agents prioritized in our pharmacovigilance analysis (Supplementary Table S7) was assembled. For each sample, a drug-target gene-set score was computed as the ssGSEA enrichment score of this 24-gene set (GSVA package). To assess robustness, the drug-target gene-set score was recomputed under leave-one-gene-out removal of each of the 24 genes and using three gene-set scoring methods (ssGSEA, GSVA and singscore). The inverse correlation with Mono_IFN abundance was stable to single-gene removal (Spearman R = −0.62 to −0.81; full set −0.75) and concordant across the three enrichment-magnitude methods (ssGSEA −0.75, GSVA −0.55, singscore −0.61) (Supplementary Figure S2).

Weighted gene co-expression network analysis (WGCNA) was performed to identify gene modules associated with these traits. To approximate scale-free network topology, the soft-thresholding power was empirically set to 16. An unsigned co-expression network was constructed, followed by transformation into a topological overlap matrix (TOM) to capture network connectivity.

Gene modules were identified using the dynamic tree cut algorithm with a minimum module size of 30 genes, and similar modules were merged using a cut height threshold of 0.25. Module eigengenes (MEs) were then computed for each module, and their associations with ssGSEA-derived scores and sepsis severity were evaluated using Pearson correlation analysis. This integrative approach enabled the identification of key co-expression modules potentially underlying the breakdown of immune homeostasis across cellular and transcriptomic scales.

Topological hub ranking (cytoHubba) was used solely to prioritize candidate genes for classifier input. The candidate feature space comprised the module-level hub genes (the intersection of the MCC, MNC, EPC and Degree rankings within the pink, greenyellow and black modules) together with the interferon-γ receptors IFNGR1 and IFNGR2, which were retained *a priori* given their central role in the single-cell IFN-γ communication analysis, yielding 33 candidate genes.

### 2.9 Ensemble machine learning framework and diagnostic feature extraction

To develop a sepsis diagnostic model with high cross-platform robustness, we implemented a large-scale ensemble machine learning framework comprising 112 algorithmic combinations. Feature selection (FS) was first performed in the training cohort (GSE65682) using seven complementary strategies: Boruta, support vector machine–recursive feature elimination (SVM-RFE), random forest variable importance (RF importance), LASSO regression, ridge regression, elastic net, and a baseline approach retaining all candidate genes (no feature selection).

Each of the seven feature sets was subsequently used as input for 16 classification algorithms. These included random forest, linear discriminant analysis (LDA), support vector machines with radial basis function kernels (SVM-Radial), k-nearest neighbors (KNN), and generalized linear models (GLM), as well as penalized regression models implemented via GLMNET with the mixing parameter α ranging from 0 to 1 in increments of 0.1, thereby spanning ridge to LASSO regularization.

All 112 model combinations were trained using 5-fold cross-validation within the training cohort for hyperparameter tuning. To rigorously assess generalizability, each trained model was further evaluated in a blinded manner across five independent external validation cohorts without retraining. Model performance was ranked based on the mean area under the receiver operating characteristic curve (AUC) across validation datasets, and the best-performing model was selected accordingly.

Finally, features with non-zero coefficients in the optimal model were extracted to construct a parsimonious diagnostic signature.

### 2.10 Target crosstalk analysis and pharmacological correlation network

To elucidate the molecular crosstalk between the diagnostic signature and high-risk drug targets, genome-wide expression profiles of the signature genes and 24 drug repurposing targets were extracted. Pairwise associations between these gene sets were quantified using Spearman’s rank correlation coefficients.

To incorporate model interpretability, feature weights derived from the optimal machine learning model (indicating positive or negative contributions to prediction) were integrated as row annotations. This enabled contextualization of gene–gene correlations within the predictive framework.

The results were visualized using hierarchical clustering heatmaps implemented in the *ComplexHeatmap* package, providing a global view of co-expression patterns. This integrative analysis facilitated the identification of key pharmacologically relevant bridging nodes exhibiting strong co-expression connectivity and potential structural targetability IFITM3.

### 2.11 Structural preparation and molecular docking

Molecular docking and reverse pharmacology screening were performed using the AutoDock Vina engine. The three-dimensional structure of the target protein IFITM3 was obtained from the AlphaFold Protein Structure Database (AlphaFold DB: AF-D3YGT6-F1), accessed via the RCSB PDB portal.

Receptor preparation was carried out using AutoDockTools, including removal of water molecules and heteroatoms, addition of missing polar hydrogens, merging of non-polar hydrogens, and assignment of Gasteiger partial charges.

The small-molecule ligand riociguat (CID: 625115-55-1) was retrieved from the PubChem database. Its three-dimensional structure was energy-minimized using OpenBabel under the MMFF94 force field. The ligand was subsequently processed in AutoDockTools, where Gasteiger charges were assigned and all rotatable bonds were defined to enable flexible docking.

For docking simulations, the grid box was centered on a putative hydrophobic pocket on the protein surface, with dimensions set to fully encompass the predicted binding region. The exhaustiveness parameter was set to 32 to enhance conformational sampling depth. Binding affinities (ΔG) for each docking pose were calculated using the Vina empirical scoring function.

The top-ranked binding pose with the lowest predicted binding energy was selected for downstream analysis. Protein–ligand interactions, including hydrogen bonds, hydrophobic contacts, and π–π stacking, were visualized and characterized at residue-level resolution using PyMOL and Discovery Studio Visualizer.

### 2.12 All-atom molecular dynamics simulation

To examine the dynamic behavior of the predicted ligand–protein complex under the selected aqueous simulation conditions, all-atom molecular dynamics simulations were performed using GROMACS. The system was parameterized using the CHARMM36 all-atom force field, while the topology of the ligand riociguat was generated using the CHARMM General Force Field (CGenFF).

The protein–ligand complex was solvated in a periodic dodecahedral (or cubic) simulation box, with a minimum distance of 1.0 nm between the protein surface and the box boundary. The system was filled with explicit TIP3P water molecules. To neutralize the system and mimic physiological ionic strength (0.15 M), appropriate numbers of Na^+^ and Cl^−^ ions were added by replacing water molecules.

Prior to production simulation, the system underwent a three-step equilibration protocol. First, energy minimization was performed using the steepest descent algorithm until the maximum force fell below 1,000.0 kJ/mol/nm, thereby removing steric clashes. Second, a 100 ps NVT equilibration was conducted under position restraints, with temperature maintained at 300 K using the V-rescale thermostat. Third, a 100 ps NPT equilibration was performed under position restraints, maintaining a pressure of 1 bar using the Parrinello–Rahman barostat.

Following equilibration, position restraints on heavy atoms were released, and a 100 ns production MD simulation was carried out under NPT conditions. The integration time step was set to 2 fs. All covalent bonds involving hydrogen atoms were constrained using the LINCS algorithm. Long-range electrostatic interactions were calculated using the Particle Mesh Ewald (PME) method with a cutoff of 1.2 nm, and van der Waals interactions were also treated with a cutoff of 1.2 nm.

Trajectory analyses were performed using built-in GROMACS tools to extract key structural and dynamic parameters, including root mean square deviation (RMSD), root mean square fluctuation (RMSF), radius of gyration (Rg), solvent-accessible surface area (SASA), and the number of intermolecular hydrogen bonds. These metrics were used to comprehensively characterize ligand-induced conformational stabilization and dynamic behavior of the target protein.

## 3 Results

### 3.1 Real-world pharmacovigilance and machine learning identify sepsis-reporting signals associated with PAH-Targeted therapies

A total of 68,052 reports containing the MedDRA Preferred Term “Sepsis” were included in the final analysis. As shown in Table 1, the sex distribution was relatively balanced, with 29,374 (43.16%) males and 29,885 (43.92%) females, while 8,793 reports (12.92%) had unspecified sex. The median age was 63 years (IQR: 50–72), although 31.19% of reports lacked age information. Body weight was also incompletely reported, with missing values in 71.24% of reports. These missing data highlight an important limitation of FAERS and should be considered when interpreting adjusted and stratified analyses.

**TABLE 1:** Report of baseline information summary table.

Characteristics	Count (n = 68,052)	Percent (%)	Median (IQR)
Sex			
Male	29,374	43.16	​
Female	29,885	43.92	​
Unknown	8,793	12.92	​
Age	​	​	63 (50–72)
<18	2,679	3.94	​
18–44	6,738	9.89	​
45–64	17,278	25.39	​
≥65	20,129	29.59	​
Unknown	21,228	31.19	​
Weight	​	​	72.27 (59.0–88.4)
<50	2,664	3.91	​
50–70	6,416	9.43	​
70–90	6,116	8.99	​
≥90	4,377	6.43	​
Unknown	48,479	71.24	​
Country			
US	30,907	45.42	​
GB	4,956	7.28	​
CA	4,466	6.56	​
JP	3,905	5.74	​
DE	3,457	5.08	​
FR	2,606	3.83	​
Other country	15,572	22.78	​
Country not specified	2,183	3.21	​
Year			
2014	2,413	3.55	​
2015	5,704	8.38	​
2016	5,910	8.68	​
2017	6,438	9.46	​
2018	7,486	11	​
2019	7,599	11.17	​
2020	8,013	11.77	​
2021	6,841	10.05	​
2022	6,605	9.71	​
2023	7,013	10.31	​
2024	4,030	5.92	​

**FIGURE 1:**
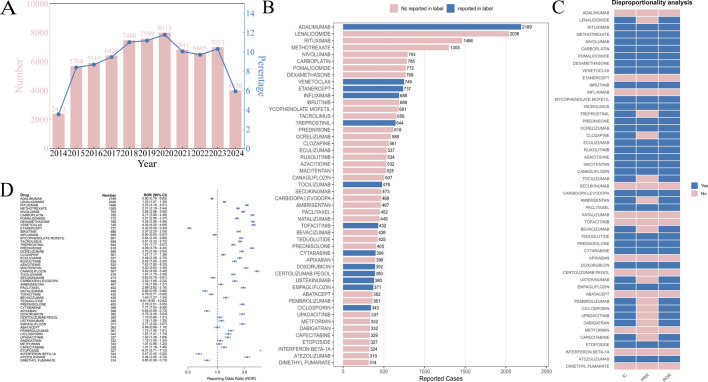
Systematic pharmacovigilance mining of the FAERS database identifies drug–sepsis associations at the population level. **(A)** Temporal trend of sepsis-associated adverse event reports within the FAERS database from 2014 to 2024. The bar chart (left y-axis) indicates the absolute number of reports per year; the line graph (right y-axis) denotes the proportion of sepsis reports relative to total adverse events. **(B)** Horizontal bar chart displaying the top 50 drugs ranked by sepsis-associated reported case counts. Blue bars indicate cases where sepsis was documented on the product label; pink bars indicate cases where sepsis was not listed on the label. Numbers adjacent to each bar represent the total case count. **(C)** Heatmap summarizing disproportionality signal detection results across three algorithms—information component (IC), proportional reporting ratio (PRR), and reporting odds ratio (ROR) — for each of the top 50 drugs. Blue cells denote a positive disproportionality signal; pink cells denote non-significant signals. **(D)** Forest plot of ROR point estimates with 95% confidence intervals for all 50 drugs. Drugs with the lower bound of the 95% CI exceeding 1.0 are considered to have a statistically significant disproportionality signal.

To investigate drug-event reporting patterns rather than causal drug effects, we conducted large-scale pharmacovigilance analyses using the FAERS database. Time-series analysis revealed a steady increase in sepsis-related adverse-event reports from 2014 to 2019, followed by a peak between 2019 and 2020 and persistently elevated reporting thereafter (Figure 1A). Baseline assessment of reporting frequencies and disproportionality metrics confirmed the expected predominance of classical immunomodulatory and antineoplastic agents among drugs frequently reported with sepsis (Figures 1B,C).

After excluding these well-established drug classes, further disproportionality analysis identified several PAH-targeted therapies with positive sepsis-reporting signals. Specifically, Maitentan, ambrisentan, and treprostinil were associated with disproportionate reporting of sepsis (Figure 1D). These results indicate reporting associations in a spontaneous adverse-event database and should not be interpreted as estimates of causal sepsis risk.

**FIGURE 2:**
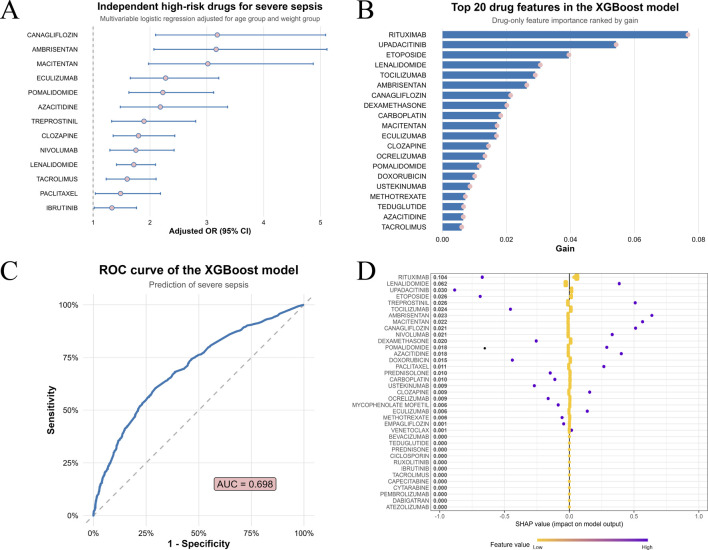
Multivariable logistic regression and XGBoost machine learning identify sepsis-reporting associations for endothelin receptor antagonists. **(A)** Forest plot of adjusted reporting odds ratios with 95% confidence intervals from multivariable logistic regression models adjusted for age group and body-weight group, displaying drugs associated with reports containing the MedDRA Preferred Term “Sepsis”. **(B)** Bar chart of the top 20 drug features ranked by information gain within the XGBoost gradient boosting model. **(C)** Receiver operating characteristic curve for the XGBoost model classifying sepsis reports, with the area under the curve annotated. **(D)** SHAP beeswarm plot illustrating the direction and magnitude of each drug feature’s contribution to model output. These analyses support reporting-pattern detection and should not be interpreted as evidence of causal drug-induced sepsis.

To further evaluate whether these reporting signals persisted after adjustment for available demographic variables, multivariable logistic regression models were constructed. After adjustment for age group and body-weight group, Maitentan and ambrisentan retained adjusted reporting associations with sepsis reports. Because FAERS lacks key clinical variables such as PAH severity, hospitalization status, catheter exposure, infection history, comorbidities, concomitant prostacyclin therapy, immunosuppressive exposure, and detailed polypharmacy, these adjusted estimates cannot establish independent causal risk factors (Figure 2A).

We also implemented an XGBoost-based model to explore nonlinear patterns associated with sepsis reporting (Figures 2B,C). Feature-importance ranking and SHAP-based attribution analyses showed that PAH-targeted therapies contributed to model classification of sepsis reports (Figure 2D). However, these machine learning results should be interpreted as algorithmic support for reporting-pattern detection, not as evidence that ERAs induce sepsis or directly increase biological susceptibility.

**FIGURE 3:**
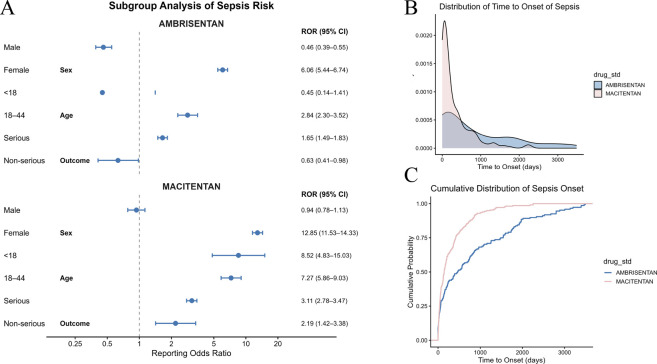
Sex-stratified and temporal patterns of sepsis reporting associated with PAH-targeted therapies. **(A)** Forest plots of reporting odds ratios (ROR) with 95% confidence intervals for ambrisentan (upper panel) and Maitentan (lower panel), stratified by sex (male vs. female), age group (<18 vs. 18–44 years), and outcome severity (serious vs. non-serious). The dashed vertical line at ROR = 1 indicates the null reference. **(B)** Kernel density estimation plot of the distribution of time to onset (TTO, in days) for sepsis events associated with ambrisentan (blue) and Maitentan (pink). **(C)** Cumulative distribution function of TTO for both drugs, illustrating the proportion of sepsis events occurring within successive time windows. Maitentan displays a steeper early accumulation curve relative to ambrisentan, consistent with a more rapid onset profile. Statistical significance was assessed using the chi-squared test for disproportionality and the Kolmogorov–Smirnov test for distributional comparison.

Subgroup analyses revealed sex-stratified heterogeneity in sepsis-reporting signals, with higher RORs for Maitentan and ambrisentan among female reports. However, this observation should be interpreted cautiously. PAH has a known female-predominant epidemiological structure, and FAERS does not provide reliable sex-stratified drug-exposure denominators, treated-population structure, PAH severity, or clinical background information. Therefore, the higher RORs observed in female reports may reflect the underlying sex distribution of ERA-treated PAH populations, differential reporting patterns, or unmeasured clinical confounding rather than true sex-specific biological susceptibility to ERA-associated sepsis. Accordingly, we interpret this result as sex-stratified reporting heterogeneity rather than evidence of a female-specific causal effect (Figure 3A).

Time-to-onset analyses showed that sepsis reports associated with ERAs were concentrated within an early reporting window, particularly within the first 100 days for Maitentan. This early-onset reporting pattern may help guide future clinical surveillance and hypothesis generation, but it does not establish a temporal causal relationship in the absence of controlled exposure, denominator, and clinical outcome data (Figures 3B,C).

### 3.2 Single-cell transcriptomic profiling identifies a sepsis-associated interferon-responsive Mono_IFN state

**FIGURE 4:**
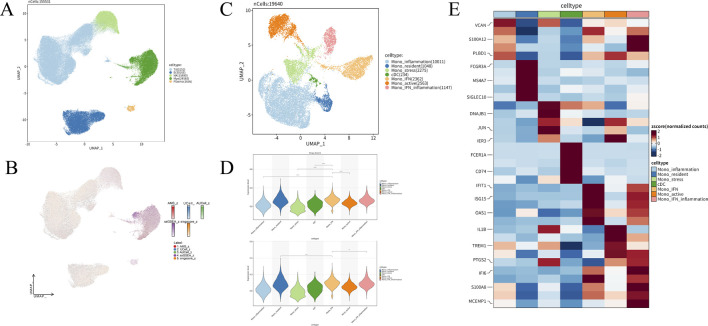
Single-cell transcriptomic profiling resolves seven functionally distinct monocyte subpopulations in sepsis peripheral blood. **(A)** UMAP projection of 155,531 peripheral blood mononuclear cells (PBMCs) colored by major cell lineage (T cells, B cells, NK cells, myeloid cells, and plasma cells), with cell counts annotated in the legend. **(B)** UMAP visualization of myeloid cell scoring using five independent gene set scoring algorithms (AMS_z, UCell_z, AUCell_z, ssGSEA_z, singscore_z), confirming concordant myeloid identity assignment. **(C)** High-resolution UMAP re-clustering of 19,640 myeloid cells into seven subpopulations: Mono_inflammation, Mono_resident, Mono_stress, cDC, Mono_IFN, Mono_active, and Mono_IFN_inflammation. Cell counts for each subpopulation are indicated in parentheses. **(D)** Violin plots comparing drug perturbation scores (upper panel) and sepsis signature scores (lower panel) across the seven monocyte subpopulations. Brackets with asterisks indicate statistically significant pairwise comparisons. **(E)** Heatmap of z-score–normalized expression of the top marker genes for each monocyte subpopulation. Columns are grouped by subpopulation identity (color bar, top); rows display representative markers including VCAN, S100A12, FCGR3A, IFIT1, ISG15, OAS1, IL1B, TREM1, and S100A8. Statistical significance was determined using the Wilcoxon rank-sum test. *P < 0.05, **P < 0.01, ***P < 0.001.

Using single-cell RNA sequencing (scRNA-seq) data from peripheral blood mononuclear cells (PBMCs), we constructed a comprehensive immune cell atlas via UMAP-based dimensionality reduction and unsupervised clustering, encompassing major immune populations including T cells, B cells, and myeloid cells (Figure 4A). Multi-dimensional single-sample gene set enrichment analyses (AddmoduleScore, AUCell, UCell, singscore and ssGSEA) revealed pronounced functional heterogeneity within the myeloid compartment (Figure 4B).

To further resolve monocyte diversity, refined subclustering delineated distinct subsets, including Mono_inflammation, Mono_resident, Mono_IFN, and cDC populations (Figure 4C, Supplementary Material S6). Comparative functional scoring (Figure 4D) and marker gene heatmaps (Figure 4E) clearly defined the transcriptional boundaries of these subsets. Notably, the Mono_IFN and related inflammatory subsets exhibited marked upregulation of interferon-stimulated genes (ISGs), including ISG15, IFIT1, and OAS1, consistent with a highly activated interferon-responsive state. These findings position Mono_IFN as a key pro-inflammatory myeloid population within the early immune landscape of sepsis.

**FIGURE 5:**
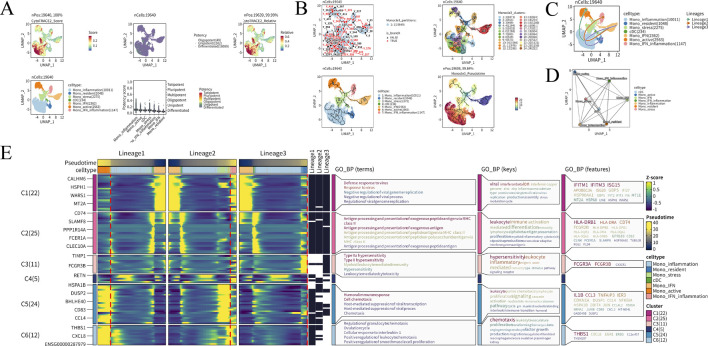
CytoTRACE stemness estimation and pseudotemporal trajectory analysis position Mono_IFN at a critical differentiation branchpoint. **(A)** CytoTRACE analysis of monocyte subpopulations. Upper panels: UMAP projections colored by CytoTRACE score (left), CytoTRACE relative score (center), and inferred developmental potency (right; Oligopotent, Unipotent, Differentiated). Lower left: UMAP colored by subpopulation identity. Lower right: box plot comparing potency scores across subpopulations, showing Mono_IFN at an intermediate differentiation state. **(B)** Monocle3 trajectory inference. Upper left: partition graph overlaid on the UMAP embedding with node connectivity. Upper right: UMAP colored by Monocle3 cluster identity. Lower left: UMAP colored by subpopulation labels. Lower right: pseudotime projection, with darker shading indicating later pseudotime values. **(C)** UMAP projection colored by three inferred differentiation lineages (Lineage 1, 2, 3), with Mono_IFN distributed across multiple trajectory branches. **(D)** PAGA (partition-based graph abstraction) connectivity map illustrating the topological relationships among subpopulations, with edge thickness proportional to connectivity strength. **(E)** Pseudotime-ordered heatmap of dynamically regulated genes organized into six co-expression clusters (C1–C6) across the three lineages. GO biological process enrichment terms, keyword clouds, and top feature genes are annotated for each cluster. C1 is enriched for antiviral defense and interferon response (IFITM1, IFITM3, ISG15); C2 for MHC class II antigen presentation (HLA-DRB1, HLA-DRA, CD74); C5 for leukocyte chemotaxis and proliferative signaling (IL1B, CCL3, TNFAIP3). Statistical significance for pseudotime-associated gene expression was determined using the Moran’s I spatial autocorrelation test.

To investigate the dynamic relationships and state transitions among monocyte subsets, we integrated CytoTRACE-based differentiation potential analysis with Monocle3 pseudotime trajectory inference. CytoTRACE scoring (Figure 5A) quantified a gradient of differentiation states across monocytes in the septic microenvironment. Trajectory reconstruction and lineage topology (Figures 5B–D) revealed a multi-branch developmental landscape, with a pronounced directional shift toward the Mono_IFN state under septic conditions.

Dynamic gene expression analysis along pseudotime, coupled with gene clustering and Gene Ontology enrichment (Figure 5E), demonstrated a progressive upregulation of interferon-related modules, prominently featuring IFITM1 and IFITM3, as cells transitioned toward the terminal Mono_IFN state. Concurrently, key antigen presentation pathways, including genes such as HLA-DRA and CD74, underwent coordinated remodeling.

Collectively, these results identify Mono_IFN as a sepsis-associated interferon-responsive monocyte state characterized by elevated interferon-stimulated genes and dynamic changes across disease states. Because the analyzed scRNA-seq datasets were derived from sepsis patients and controls rather than ERA-exposed PAH patients, these findings should be interpreted as describing sepsis-related monocyte-state heterogeneity and should not be taken as evidence that ERAs induce Mono_IFN polarization.

### 3.3 Cell–cell communication, temporal dynamics, and Co-expression networks reveal multiscale breakdown of immune homeostasis

**FIGURE 6:**
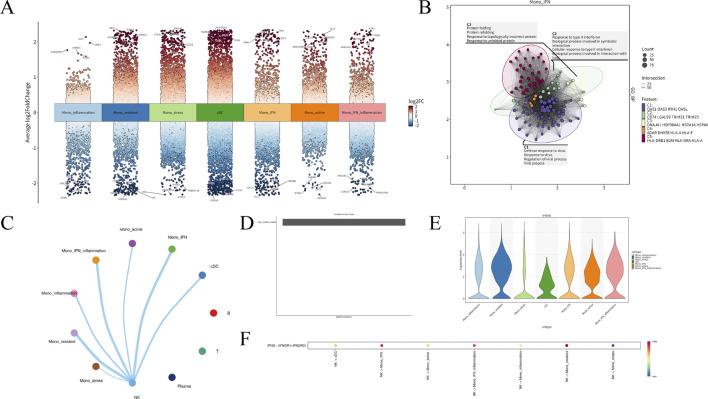
Mono_IFN functions as a central interferon signaling hub within the sepsis intercellular communication network. **(A)** Strip chart displaying differentially expressed genes (DEGs) for each monocyte subpopulation between sepsis and healthy control conditions. Each dot represents a gene; color encodes log_2_ fold change (red = upregulated, blue = downregulated). Representative gene names are annotated. **(B)** Network visualization of Gene Ontology biological process (GO_BP) enrichment for Mono_IFN DEGs, organized into five functional clusters (C1–C5). C1: defense response to virus and regulation of viral processes (OAS1, OAS3, IFIH1, OASL); C2: response to type II interferon; C3: protein refolding and unfolded protein response; C4: ADAR-mediated RNA editing and MHC class I presentation; C5: MHC class II antigen processing (HLA-DRB1, B2M, HLA-DRA). Node size represents gene count; edge thickness represents shared gene overlap. **(C)** CellChat-inferred intercellular communication network among all cell types. Node size is proportional to interaction strength; edge color and width encode the directionality and magnitude of signaling. Mono_IFN_inflammation and Mono_stress occupy positions with highest outgoing and incoming interaction weights, respectively. **(D)** Bar plot quantifying the relative contribution of the IFN-γ (IFNG)–IFNGR1/IFNGR2 ligand–receptor pair to total intercellular communication. **(E)** Violin plot of IFNGR2 expression levels across all seven monocyte subpopulations. **(F)** Dot plot showing the probability and directionality of IFNG–IFNGR1/IFNGR2 signaling from NK cells to each monocyte subpopulation and cDCs. Color intensity encodes communication probability; dot presence indicates statistical significance. Statistical significance for differential expression was determined using the Wilcoxon rank-sum test (Bonferroni-adjusted P < 0.05).

To systematically characterize the molecular functions and microenvironmental interactions of the polarized Mono_IFN subpopulation, we first identified differentially expressed genes (DEGs) across cell subsets (Figure 6A). Gene Ontology (GO) network clustering of Mono_IFN-associated genes (Figure 6B) revealed significant enrichment in antiviral defense, unfolded protein response, and type II interferon signaling pathways, supporting a strongly activated inflammatory phenotype.

At the level of intercellular communication, network topology analysis and quantitative assessment of ligand–receptor contributions (Figures 6C,D) consistently demonstrated that Mono_IFN occupies a central role as a major signal-receiving hub within the septic microenvironment. In particular, the IFN-γ signaling axis—primarily originating from natural killer (NK) cells via IFNG–(IFNGR1/IFNGR2)—was markedly enriched (Figure 6F), with IFNGR2 exhibiting high transcriptional expression in Mono_IFN (Figure 6E). These findings suggest that Mono_IFN functions not only as a terminal polarization state but also as a key effector population that integrates upstream immune signals and amplifies systemic inflammatory cascades.

**FIGURE 7:**
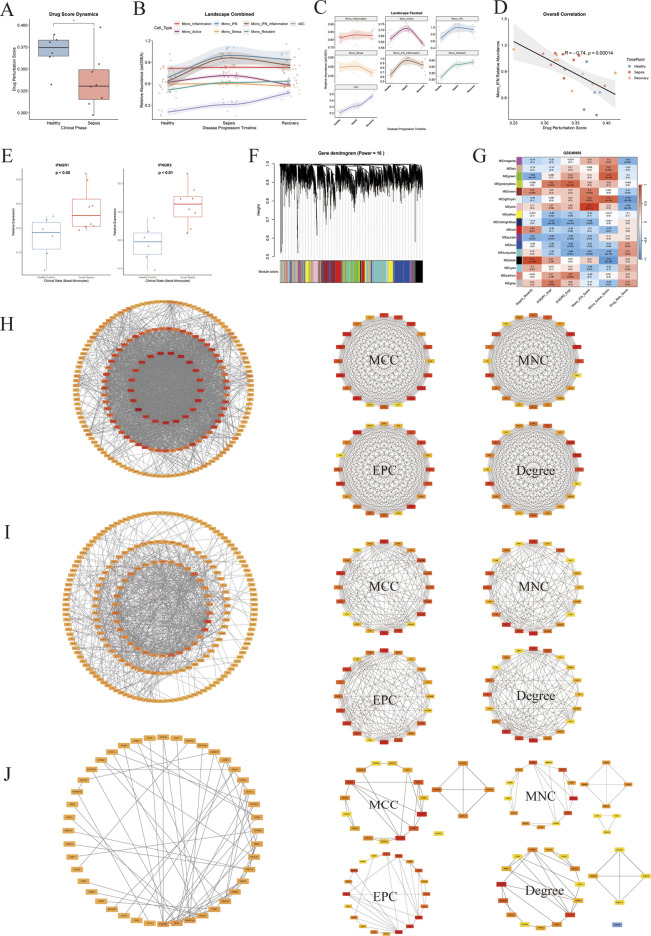
Integration of drug perturbation scoring, single-cell dynamics, and WGCNA identifies core gene modules linking ERA exposure to Mono_IFN-driven immunodysregulation. **(A)** Box plot comparing drug perturbation scores between healthy controls and sepsis patients. **(B)** Line plot depicting the relative abundance trajectory of each monocyte subpopulation across disease progression stages (Healthy → Sepsis → Recovery). Mono_IFN (green) displays a transient expansion peaking during the sepsis phase. **(C)** Faceted line plots showing individual subpopulation dynamics. **(D)** Scatter plot demonstrating the inverse correlation between drug perturbation score and Mono_IFN fractional abundance (Spearman R = −0.74, P = 0.00014). **(E)** Box plots of IFNGR1 (left) and IFNGR2 (right) bulk expression levels comparing healthy controls with acute sepsis patients. **(F)** WGCNA gene dendrogram and module assignment at soft-thresholding power = 16. **(G)** Module–trait correlation heatmap, displaying Pearson correlation coefficients and significance levels between module eigengenes (rows) and clinical/cellular traits (columns). **(H)** Full gene co-expression network of the pink module, which is most highly correlated with Mono_IFN abundance, with hub gene subnetworks extracted via the cytoHubba plugin in Cytoscape using four topological algorithms: MCC, MNC, EPC, and degree centrality. **(I)** Equivalent network analysis for the black module, which is most correlated with drug perturbation score. **(J)** Refined hub gene interaction networks for the greenyellow intersection module, utilizing cytoHubba-derived four-algorithm cross-validation to identify the final candidate gene set. Statistical significance for module–trait correlations was assessed using Student’s asymptotic P-values for Pearson correlation. *P < 0.05, **P < 0.01.

To further delineate the temporal dynamics of this population during disease progression, we analyzed changes in relative cell abundance across healthy, septic, and recovery states. The resulting landscape (Figures 7B,C) revealed a pronounced, unimodal expansion of the Mono_IFN subset during the acute phase of sepsis. Elevated expression of IFNGR1 and IFNGR2 in this subset was independently validated in external clinical cohorts of acute sepsis patients (Figure 7E), supporting the reproducibility of this signaling axis.

To bridge cellular phenotypes with system-level immune states, we computed a drug-target gene-set score (ssGSEA of the 24 PAH drug-target genes; Methods). This score was lower in patients with sepsis than in healthy controls (Figure 7A) and was inversely correlated with the relative abundance of Mono_IFN (Spearman R = −0.74, p = 0.00014; Figure 7D). This inverse association was robust to leave-one-gene-out removal and concordant across three enrichment-based scoring methods (Supplementary Figure S2).

To further identify molecular drivers underlying this multiscale dysregulation, we constructed a weighted gene co-expression network (Figure 7F; Supplementary Figure S2). Module–trait correlation analysis (Figure 7G) identified key co-expression modules MEblack strongly associated with Mono_IFN abundance, drug perturbation scores, and sepsis severity. Hub genes within these modules were subsequently prioritized using multiple topological algorithms, including MCC, MNC, EPC, and Degree (Figures 7H–J).

Collectively, these analyses delineate a coordinated network linking drug-induced perturbations to immune dysregulation at both cellular and transcriptomic levels, and establish a robust feature space for downstream machine learning–based diagnostic modeling.

These analyses should be interpreted as hypothesis-generating associations between PAH drug-target gene-set variation and sepsis-associated interferon-responsive monocyte features. They do not establish that ERA exposure induces the Mono_IFN state. In addition, because IFITM3, ISG15, IFIT1, OAS1, and related interferon-stimulated genes are broadly activated across viral infection, sepsis, and systemic inflammation, the observed Mono_IFN program should not be considered specific to ERA-associated sepsis reporting.

### 3.4 An ensemble machine learning framework identifies a robust diagnostic signature for sepsis

Given that early immune dysregulation in sepsis arises from highly complex, network-level transcriptomic perturbations, single biomarkers are often insufficient for accurate clinical prediction. To systematically capture these high-dimensional features, we developed an ensemble machine learning framework comprising 112 algorithmic combinations, integrating multiple feature selection strategies with diverse classifiers (Supplementary Figure S3).

**FIGURE 8:**
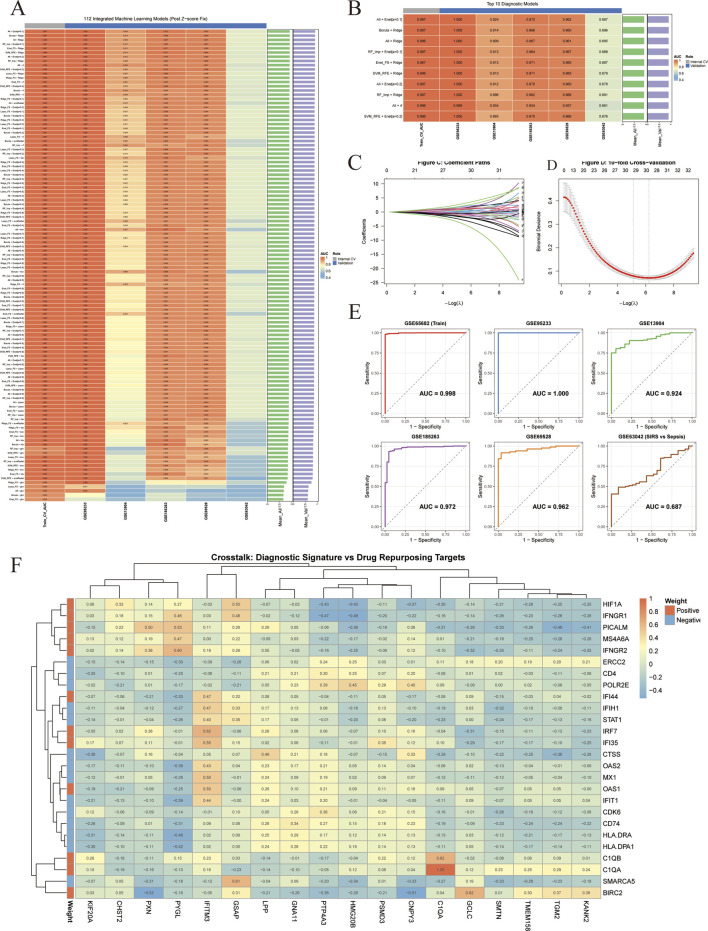
A 112-algorithm ensemble machine learning framework constructs a multi-gene diagnostic signature for sepsis classification. **(A)** Heatmap of AUC performance across 112 machine learning algorithm combinations (rows) evaluated on the training set (GSE65682) and five independent external validation cohorts (columns). All cohorts underwent within-cohort Z-score normalization followed by rank transformation to ensure cross-platform comparability. Color scale ranges from blue AUC=0.5 through yellow to red AUC=1.0 . Right-side bar plots display the mean AUC across all validation cohorts (Mean_Val, purple) and across all cohorts including training (Mean_All, green). **(B)** Detailed AUC performance of the top 10 diagnostic models selected by mean validation AUC. Each cell displays the exact AUC value. The top annotation bar distinguishes internal cross-validation (gray) from external validation cohorts (blue). **(C)** Coefficient trajectory plot showing the regularization path of gene features as a function of −log(λ) in the elastic net model corresponding to the top-ranked algorithm combination. Each curve represents one gene; coefficients shrink toward zero as the penalty increases. **(D)** Binomial deviance plot from 10-fold cross-validation of the elastic net model. The left dashed line indicates the λ at minimum deviance (lambda.min) and the right dashed line indicates the λ within one standard error (lambda.1se). The optimal model was selected at lambda. min. **(E)** Receiver operating characteristic (ROC) curves for the final diagnostic signature evaluated on the training cohort and five external validation cohorts. The training cohort (GSE65682) contained sepsis patients and healthy controls. Among the validation cohorts, GSE95233, GSE13904, GSE185263, and GSE69528 each compared sepsis patients with healthy controls, while GSE63042 compared sepsis patients with systemic inflammatory response syndrome (SIRS) patients, representing a more clinically challenging discrimination task. AUC values are displayed within each panel. **(F)** Spearman correlation heatmap depicting the crosstalk between diagnostic signature genes (rows) and candidate drug-repurposing targets derived from FAERS pharmacovigilance analysis (columns). Cell values represent correlation coefficients computed on the full GSE65682 expression matrix. The left annotation bar indicates the sign of each gene’s weight in the diagnostic model (red = positive weight, indicating upregulation in sepsis; blue = negative weight, indicating downregulation). Hierarchical clustering was applied to both rows and columns to reveal co-regulation patterns between diagnostic markers and therapeutic targets.

Model training was conducted using rigorous internal cross-validation within the training cohort (GSE65682), followed by parallel evaluation of all models across five fully independent external validation cohorts (GSE95233, GSE13904, GSE185263, GSE69528, and GSE63042) (Figure 8A). Notably, while four validation cohorts compared sepsis patients against healthy controls, GSE63042 compared sepsis patients against systemic inflammatory response syndrome (SIRS) patients, representing a more clinically stringent discrimination task. All cohorts underwent independent within-cohort Z-score normalization and rank transformation prior to model training to ensure cross-platform comparability. This design minimized dataset-specific overfitting and enabled comprehensive assessment of model generalizability across heterogeneous platforms, populations, and clinical settings.

Global benchmarking of model performance (Figure 8B) revealed that an elastic net–regularized regression model using all input features All+Enet,α=0.1 achieved the highest and most stable mean area under the curve (AUC) across validation cohorts. Optimization of the penalization parameter based on binomial deviance (Figures 8C,D) resulted in a parsimonious model retaining 29 non-zero coefficient genes, thereby defining a multi-gene diagnostic signature.

Receiver operating characteristic (ROC) analysis (Figure 8E) demonstrated near-perfect predictive performance in the training set and consistently high accuracy across the four validation cohorts employing healthy controls. As expected, the model achieved a comparatively lower but still discriminative AUC on the GSE63042 cohort, consistent with the inherently greater molecular overlap between sepsis and SIRS relative to that between sepsis and healthy states. These results indicate that the diagnostic signature robustly captures transcriptomic dysregulation associated with acute sepsis across diverse datasets, and retains meaningful discriminatory capacity even in the more challenging sepsis-versus-SIRS context.

Importantly, this model provides a quantitative framework for prioritizing biologically relevant features within the signature, thereby enabling downstream identification of candidate genes with potential translational and therapeutic relevance.

### 3.5 Molecular docking and dynamics simulations generate an exploratory Riociguat–IFITM3 interaction hypothesis

To explore whether IFITM3 may contain a computationally identifiable small-molecule interaction site, we performed exploratory molecular docking using an FDA-approved small-molecule library (Supplementary Material S7). The IFITM3 structural model was obtained from AlphaFold DB; therefore, the docking results should be interpreted cautiously, particularly because IFITM3 is a small membrane-associated protein and the predicted structure may not fully represent its native membrane-embedded conformation.

**FIGURE 9:**
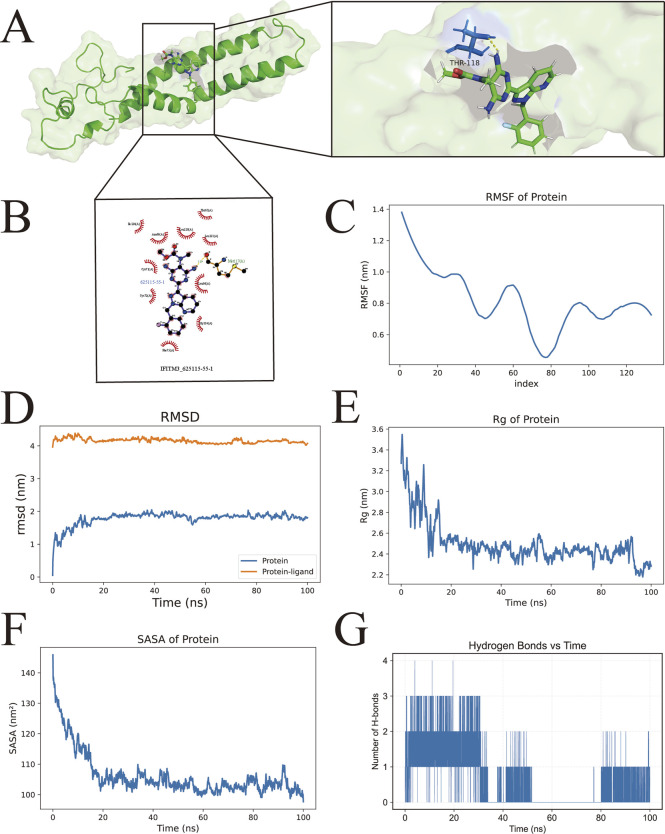
Exploratory molecular docking and aqueous MD simulation of the predicted riociguat–IFITM3 complex. **(A)** Three-dimensional ribbon representation of the IFITM3 protein structure (green) with riociguat (blue sticks) docked at the predicted binding pocket. The inset provides a magnified view of the binding interface, highlighting the key interacting residue THR-118 and the surrounding molecular surface. **(B)** Two-dimensional ligand interaction diagram of the riociguat–IFITM3 complex (compound ID: 625115-55-1), showing hydrogen bonds, hydrophobic contacts, and van der Waals interactions with specific residues. Red semicircular arcs denote hydrophobic interactions; dashed lines indicate hydrogen bonds. **(C)** Root mean square fluctuation (RMSF) profile of IFITM3 residues over the 100-ns simulation trajectory, reflecting per-residue flexibility. Lower RMSF values in the binding region indicate ligand-induced structural stabilization. **(D)** Root mean square deviation (RMSD) of the protein backbone (blue) and the protein–ligand complex (orange) over the 100-ns simulation. The protein–ligand complex maintains a stable RMSD plateau (∼4.2 nm) from approximately 20 ns onward, confirming conformational convergence. **(E)** Radius of gyration (Rg) of the IFITM3 protein over the simulation trajectory, stabilizing around 2.4 nm after initial equilibration, indicating maintenance of overall structural compactness. **(F)** Solvent-accessible surface area (SASA) of IFITM3 over the 100-ns trajectory, converging to approximately 100 nm^2^ after initial compaction. **(G)** Time-resolved hydrogen bond count between riociguat and IFITM3 over the full 100-ns trajectory. The complex sustains 1–3 intermolecular hydrogen bonds throughout the simulation, with transient peaks of 4 bonds, confirming persistent polar interactions.

Molecular docking nominated riociguat as a candidate compound with a favorable predicted docking score for a putative hydrophobic pocket in the IFITM3 model (predicted binding energy = −8.1 kcal/mol). Structural visualization suggested that riociguat could occupy this predicted pocket and form a hydrogen bond with Thr118, together with hydrophobic interactions and π–π stacking (Figure 9A). Interaction profiling further illustrated the predicted residue-level contact pattern between riociguat and the IFITM3 model (Figure 9B). These results indicate a computationally predicted interaction pattern and do not establish direct biochemical binding, druggability of the predicted pocket, or functional modulation of IFITM3.

To further examine the dynamic behavior of this predicted complex under the selected simulation conditions, we conducted a 100-ns all-atom molecular dynamics simulation in an aqueous, non-membrane environment. RMSD analysis showed that the simulated protein–ligand complex reached an apparent equilibrium after approximately 20 ns, whereas the apo IFITM3 model showed greater structural deviation over time (Figure 9D). Residue-level RMSF analysis indicated relatively low fluctuation across most regions of the predicted complex, with moderate mobility mainly observed in loop regions (Figure 9C). Radius of gyration analysis showed that the simulated complex remained within a relatively narrow range during the production simulation (Figure 9E), and SASA analysis suggested reduced solvent exposure under the simulation conditions (Figure 9F). Hydrogen-bond analysis showed transient formation and dissociation of ligand–protein hydrogen bonds during the trajectory, indicating a dynamic rather than permanently fixed interaction pattern (Figure 9G).

Taken together, these docking and MD analyses nominate a possible riociguat–IFITM3 structural interaction as an exploratory *in silico* hypothesis. Because the MD simulation was performed outside a lipid membrane environment, these findings cannot adequately recapitulate the native membrane context of IFITM3 and should not be interpreted as evidence of physiological target engagement. Moreover, the results do not demonstrate that riociguat binds IFITM3 in biological systems, modulates IFITM3-associated interferon signaling, affects monocyte function, or provides therapeutic protection in sepsis. Biophysical binding assays, membrane-context structural modeling, IFITM3-expressing cellular systems, monocyte-based interferon-response assays, cytokine readouts, and *in vivo* sepsis models will be required to determine whether this predicted interaction has biological relevance.

## 4 Discussion

This study presents a systematic translational framework that integrates real-world pharmacovigilance, single-cell and bulk transcriptomic network analyses, and all-atom molecular dynamics simulations within a unified analytical pipeline. By adopting this top-down systems biology strategy, we trace drug safety signals identified at the population level to cellular-scale immune perturbations and ultimately to protein–ligand interactions at atomic resolution, yielding four sequentially connected key findings.

First, leveraging large-scale FAERS data, we combined multidimensional disproportionality analyses (ROR, PRR, and IC) with XGBoost-based attribution to identify endothelin receptor antagonists (ERAs), specifically Maitentan and ambrisentan, as non-conventional drugs associated with increased sepsis risk at the population level. SHAP-based interpretation further highlighted their nonlinear contributions to risk, while subgroup and temporal analyses revealed marked sex-specific susceptibility and an early-onset risk profile.

Second, single-cell transcriptomic profiling of peripheral blood mononuclear cells identified a distinct interferon-responsive monocyte subset (Mono_IFN) as a central cellular component associated with systemic immune dysregulation. This population is characterized by elevated expression of interferon-stimulated genes, including IFITM3 and ISG15, and functions as a major signal-receiving hub within intercellular communication networks, particularly in mediating IFN-γ signaling across immune cell types.

Third, at the bulk transcriptomic level, WGCNA identified co-expression modules strongly associated with Mono_IFN abundance. Building upon this, a large-scale ensemble machine learning framework encompassing 112 algorithmic combinations yielded a robust 29-gene diagnostic signature with consistent cross-platform performance. Within this model, IFITM3 emerged as a top-weighted feature, suggesting its potential relevance as a key molecular indicator of immune dysregulation.

Finally, 100-ns all-atom molecular dynamics simulations supported stable binding between riociguat and IFITM3, with convergence of RMSD after ∼20 ns and sustained hydrogen bond interactions, indicating favorable structural compatibility. While these findings are based on computational modeling, they provide thermodynamic support for the potential repurposing of riociguat as a modulator of IFITM3-associated pathways.

Collectively, this multi-scale analysis links drug-associated risk signals to cellular and molecular mechanisms, and highlights IFITM3 as a convergent node connecting pharmacological exposure, immune dysregulation, and potential therapeutic intervention. These findings provide a conceptual framework for integrating pharmacovigilance with systems immunology to inform precision medicine strategies in sepsis.

Among the findings of this study, the observed association between PAH-targeted therapies and sepsis risk serves as the epidemiological anchor for the subsequent mechanistic investigations. From a methodological perspective, disproportionality analysis of FAERS data has been widely established as a cornerstone for early post-marketing safety signal detection, and its integration with gradient boosting approaches has been shown to substantially enhance signal sensitivity (De Abreu Ferreira et al., 2024). However, prior studies examining drug–sepsis associations have largely focused on classical immunosuppressants or antineoplastic agents, while the potential contribution of cardiovascular-targeted therapies—particularly endothelin receptor antagonists (ERAs) used in PAH—has remained largely unexplored at the population level.

From a biological standpoint, the endothelin system exhibits extensive crosstalk with the pathophysiological cascades of sepsis. Clinically, plasma endothelin-1 (ET-1) levels are markedly elevated in septic patients and correlate with myocardial injury and increased mortality. At the molecular level, ET-1 can activate NF-κB signaling via ETA receptors, driving the production of pro-inflammatory cytokines such as TNF-α and IL-6. In addition, ET-1 promotes endothelial activation through upregulation of ICAM-1 and facilitates macrophage polarization toward an M1-like inflammatory phenotype.

Importantly, endothelin signaling is not restricted to the vascular compartment: ETA and ETB receptors are broadly expressed on T and B lymphocytes as well as myeloid cells, where they modulate IFN-γ production and immune cell activation (Vuolo et al., 2015). Activated T cells can further induce endothelin-1 production in monocytes via IFN-γ and TNF-α, establishing an immune-mediated feedback loop independent of endothelial cells.

Taken together, these multilayered interactions suggest that pharmacological blockade of endothelin receptors by ERAs, while beneficial for pulmonary vascular tone regulation, may inadvertently disrupt non-canonical roles of the endothelin axis in maintaining innate immune homeostasis. In particular, interference with monocyte polarization dynamics and endothelial–immune cell communication networks may compromise host defense against systemic infection, especially within critical early exposure windows.

Notably, the pronounced sex-specific risk observed for Maitentan, with substantially elevated sepsis reporting in female patients, is consistent with the known epidemiological bias of PAH and aligns with reported sex-related heterogeneity in sepsis outcomes (GBD 2021 Global Sepsis Collaborators, 2025). Given that major clinical trials such as SERAPHIN were not specifically designed or powered to detect rare but severe infectious complications (Pulido et al., 2013), the signal identified here from large-scale real-world data provides complementary evidence that may not be captured in conventional trial settings.

The sex-stratified findings should also be interpreted within the limitations of spontaneous reporting data. Although Maitentan and ambrisentan showed higher sepsis-reporting RORs among female reports, this pattern cannot be directly translated into biological susceptibility. PAH is more common in women, and the treated population receiving ERAs may therefore be enriched for female patients. Because FAERS lacks sex-stratified exposure denominators, detailed drug-utilization distributions, PAH population structure, disease severity, hospitalization status, comorbidities, and concomitant therapies, the observed female-enriched reporting signal may be explained by population structure or reporting bias. Future studies based on prescription-linked clinical cohorts or registries will be required to determine whether any true sex-specific difference exists in infection-related outcomes among ERA-treated patients.

Collectively, these findings extend current understanding of ERA safety profiles and suggest that heightened clinical vigilance may be warranted during the early phase of ERA therapy, particularly with respect to systemic immune monitoring and infection risk assessment.

From a mechanistic perspective, the monocyte subpopulation landscape and functional heterogeneity revealed by our scRNA-seq analysis engage in a multi-layered dialogue with current paradigms in sepsis immunobiology. Sepsis is fundamentally characterized by a biphasic immune trajectory, with an early hyperinflammatory phase driven by M1-like macrophage activation, followed by a transition toward persistent immunosuppression marked by reduced HLA-DR expression, impaired antigen presentation, and skewing toward M2-like polarization (Wang and Wang, 2023).

Importantly, monocytes are not a homogeneous population along this polarization spectrum. Reyes et al. identified an MS1 monocyte state (CD14^+^HLA-DR^low^IL1R2^high^) in septic patients, characterized by elevated expression of RETN, ALOX5AP, and S100A8, functionally resembling monocytic myeloid-derived suppressor cells (M-MDSCs), and strongly associated with disease severity and mortality (Reyes et al., 2020). Subsequent studies demonstrated that plasma from patients with sepsis or severe COVID-19 can drive hematopoietic progenitors toward this MS1 program in an IL-6- and IL-10-dependent manner.

In contrast, the Mono_IFN subpopulation identified in this study exhibits a distinct transcriptional signature dominated by interferon-stimulated genes (ISGs), including IFIT1, ISG15, OAS1, and IFITM3, rather than the S100 A family and IL1R2 characteristic of MS1. This distinction suggests that Mono_IFN represents a biologically independent state within the monocyte polarization continuum—one that is not primarily immunosuppressive, but instead reflects an interferon-driven inflammatory effector phenotype. Notably, IFN-γ signaling in sepsis exhibits a well-recognized duality. On one hand, IFN-γ has been shown to reverse sepsis-induced immunoparalysis by restoring monocyte HLA-DR expression and enhancing pathogen clearance. On the other hand, sustained IFN-γ signaling and persistent ISG activation can exacerbate tissue damage: through JAK–STAT1-mediated epigenetic priming, monocytes become hyperresponsive to secondary stimuli, producing excessive levels of TNF-α and IL-6 (Mishra and Ivashkiv, 2024).

Recent large-scale integrative analyses from the SUBSPACE consortium further conceptualize sepsis immune dysregulation as two orthogonal axes—myeloid and lymphoid dysfunction—and identify a “myeloid protective cluster” enriched for interferon responses and mature innate immune cells (Chang et al., 2025). While the Mono_IFN population described here partially overlaps with this interferon-enriched program at the transcriptional level, its dynamic expansion during disease progression and its central positioning within intercellular communication networks suggest a functional shift from protective to pathological states. Supporting this notion, independent single-cell studies have reported upregulation of IFITM2, IFITM3, S100A8, and S100A9 in monocytes during the transition from high-risk states to overt sepsis, consistent with the elevated IFITM3 expression observed in our Mono_IFN subset.

Integrating these lines of evidence, Mono_IFN can be conceptualized as a monocyte substate emerging under conditions of excessive interferon signaling, characterized by both amplification of inflammatory effector functions and a central role in cross-cell-type signal propagation. This functional profile extends beyond the purely immunosuppressive paradigm represented by MS1 cells. Notably, pseudotime analysis in our study positions Mono_IFN as an intermediate transitional state along the monocyte differentiation trajectory, while cell–cell communication analysis identifies it as a dominant relay hub for IFN-γ/IFNGR signaling, bridging interactions among NK cells, cDCs, and effector monocytes. This topological configuration suggests a potential “bottleneck effect,” whereby dysregulation within the Mono_IFN compartment may disproportionately amplify global immune network instability in a nonlinear manner.

The translational implications of the above micro-scale immunological findings converge on two principal dimensions: diagnostic biomarkers and therapeutic intervention targets. At the diagnostic level, the 29-gene signature derived from a systematic evaluation of 112 integrated machine learning model combinations demonstrated robust and consistent discriminative performance across multiple independent external cohorts. Among the 24 perturbation-related genes, IFITM3 exhibited the most extensive correlation with the diagnostic signature.

As a canonical interferon-stimulated gene (ISG), IFITM3 was initially characterized for its broad antiviral activity, primarily through inhibition of viral–endosomal membrane fusion, thereby restricting the cellular entry of enveloped viruses such as influenza, dengue, and West Nile virus (Everitt et al., 2012). Genetic variation in IFITM3, particularly the rs12252 polymorphism, has been consistently associated with susceptibility to severe viral infections, including influenza and COVID-19 (Shoukat et al., 2021).

Beyond its antiviral role, IFITM3 functions as a critical regulatory node within interferon signaling networks. It exerts negative feedback on type I interferon production by promoting autophagosome-dependent degradation of IRF3 (Kearney et al., 2017), and modulates TLR-dependent cytokine responses in myeloid cells through regulation of Nogo-B turnover (Tang et al., 2022). These findings indicate that IFITM3 is not merely an antiviral effector but a key modulator of inflammatory homeostasis, whose dysregulation may contribute to uncontrolled cytokine production—a hallmark of sepsis pathophysiology. Additional roles of IFITM3 in B cell receptor signaling and PI3K pathway amplification, as well as its involvement in γ-secretase regulation in neurodegenerative contexts (Gewies et al., 2014; Gaudelli et al., 2020), further underscore its multifunctional nature at the intersection of immunity, inflammation, and disease.

Within this framework, the identification of IFITM3 as the highest-weighted feature in our diagnostic model is biologically coherent, as its expression level may serve as a proxy for the activation intensity of interferon-responsive pathways, which in turn reflect the trajectory of immune dysregulation in sepsis.

At the exploratory computational level, our molecular docking and molecular dynamics analyses generated a potential riociguat–IFITM3 interaction hypothesis. Riociguat is the first approved soluble guanylate cyclase (sGC) stimulator and exerts its established pharmacological effects by directly stimulating sGC and sensitizing it to endogenous nitric oxide (NO), thereby increasing intracellular cGMP production and producing vasodilatory, antiproliferative, and antifibrotic effects (Lian et al., 2017). The NO–sGC–cGMP axis is well established in pulmonary vascular biology (Benza et al., 2024), and alterations in NO bioavailability and sGC activity have also been implicated in sepsis-associated vascular dysfunction (Moya-Díaz et al., 2024). These established vascular effects provide a pharmacological context for considering riociguat in cardiopulmonary disease, but they do not by themselves imply direct immunomodulatory activity through IFITM3.

In the present study, 100-ns all-atom molecular dynamics simulations suggested that riociguat could remain associated with the predicted IFITM3 structural model under the simulated conditions. This was supported by convergence of RMSD, relative stability of the radius of gyration, reduced solvent-accessible surface area, and a dynamic pattern of hydrogen-bond formation and dissociation. However, these observations should be interpreted strictly as *in silico* evidence of structural compatibility. They do not establish direct biochemical binding, target engagement in cells, or functional modulation of IFITM3-mediated interferon or TLR-related signaling pathways. In particular, because IFITM3 is a membrane-associated protein and the current simulation was performed using a predicted structure outside its native membrane environment, the biological relevance of this interaction remains uncertain.

Therefore, the riociguat–IFITM3 finding should be viewed as a hypothesis-generating result rather than as evidence for a validated therapeutic mechanism. We do not infer from these data that riociguat can reverse ERA-associated sepsis susceptibility or serve as a protective intervention in patients receiving endothelin receptor antagonists. Instead, the predicted interaction provides a possible starting point for future experimental work. Direct biophysical assays, such as surface plasmon resonance or isothermal titration calorimetry, will be required to test binding affinity. Cellular assays using monocytes or IFITM3-expressing systems will be needed to evaluate whether riociguat affects IFITM3-associated interferon responses, TLR-dependent cytokine production, or inflammatory signaling. Ultimately, *in vivo* sepsis models and carefully designed clinical studies would be necessary before any immunomodulatory or protective role of riociguat could be considered.

Nevertheless, the role of interferon signaling and IFITM3 in sepsis should be interpreted in a context-dependent manner. Interferon responses are not uniformly pathogenic in sepsis. In patients with sepsis-associated immunoparalysis, recombinant interferon-γ has been reported to restore monocyte HLA-DR expression and improve *ex vivo* cytokine responsiveness, suggesting a potential immunorestorative role in selected immune-suppressed states. In contrast, excessive or persistent interferon-γ activity may define a distinct hyperinflammatory endotype. Recent clinical evidence showed that an interferon-γ/CXCL9-high sepsis endotype was independently associated with increased 28-day mortality, whereas early decreases in IFN-γ and CXCL9 levels were associated with better outcomes. These observations indicate that the biological consequence of interferon signaling depends on disease phase, immune endotype, and the balance between antimicrobial defense and inflammatory tissue injury.

A similar caution applies to IFITM3. Although IFITM3 emerged as a high-weight feature in our diagnostic model and was enriched in the Mono_IFN state, this does not establish IFITM3 as a causal driver of sepsis progression. IFITM3 is classically recognized as an interferon-stimulated antiviral restriction factor, and experimental studies have shown that it can protect the host against severe viral infection. In addition, IFITM3 has been reported to restrain virus-induced inflammatory cytokine responses through mechanisms involving TLR-related signaling regulation. Therefore, increased IFITM3 expression in sepsis may reflect activation of an interferon-responsive host-defense program, a maladaptive inflammatory state, or both, depending on the cellular and clinical context. In the present study, IFITM3 should therefore be regarded as a candidate marker and network-associated node of interferon-responsive monocyte dysregulation rather than as a validated pathogenic mediator (Everitt et al., 2012; Döcke et al., 1997; Giamarellos-Bourboulis et al., 2024; Clement et al., 2022).

This study has several limitations that warrant careful consideration. First, as a spontaneous adverse event reporting system, FAERS is inherently subject to multiple sources of bias. Underreporting is systematic, particularly for severe events such as sepsis that may be attributed to underlying disease processes. Reporting behavior is further influenced by non-random factors, including media attention, regulatory warnings, and prescribing practices. In addition, the absence of standardized information on baseline comorbidities and concomitant medications limits the ability to fully eliminate residual confounding. Although we applied multivariable regression and machine learning-based feature selection to mitigate false-positive signals, disproportionality analysis fundamentally serves as a signal detection tool, providing relative measures of association rather than establishing causality. Whether the observed association between ERAs and sepsis translates into causal risk at the interventional level requires validation in well-designed, large-scale prospective real-world cohorts. Ideally, such validation would involve systematic collection of infection-related endpoints within PAH registries and the application of time-dependent Cox regression analyses.

Several limitations of the adjusted FAERS analyses should be emphasized. Although multivariable logistic regression was used to adjust for available demographic variables, the adjusted estimates cannot establish Maitentan or ambrisentan as independent clinical risk factors for sepsis. FAERS lacks reliable information on major confounders that are highly relevant to PAH populations, including PAH severity, functional class, hospitalization status, central venous access, infection history, comorbidities, concomitant prostacyclin therapy, immunosuppressive exposure, and detailed polypharmacy. In addition, age and body weight were incompletely reported, particularly body weight, which further limits the interpretability of adjusted models. Therefore, the associations observed for Maitentan and ambrisentan should be interpreted as adjusted reporting associations within a spontaneous reporting system rather than evidence of independent causal risk. Validation in controlled clinical cohorts with drug-exposure denominators and detailed covariate adjustment is required.

Second, while our transcriptomic integration spans both single-cell and bulk RNA-seq datasets—enabling a cross-scale linkage from cellular heterogeneity to population-level diagnostic modeling—the current analysis remains constrained by the cross-sectional nature of publicly available datasets. As a result, the temporal dynamics of disease progression from early infection to overt sepsis cannot be fully resolved. In particular, the trajectory of Mono_IFN abundance and its functional state transitions across distinct clinical phases (hyperinflammatory, immunosuppressive, and recovery stages) require prospective longitudinal sampling at single-cell resolution to be accurately delineated. The co-expression network was derived from a single monocyte cohort of modest size n=22 ; module assignments are therefore exploratory and hypothesis-generating, and we do not claim cross-cohort module preservation. Topological hub status reflects network connectivity rather than demonstrated mechanism. The drug-target gene-set score is a descriptive transcriptional enrichment measure rather than a measure of drug exposure; its inverse association with Mono_IFN abundance, although robust to gene-set composition and concordant across enrichment-magnitude scoring methods, was sign-discordant under AUCell and should be interpreted as directionally suggestive. The proposed link to PAH-therapy exposure is a hypothesis derived from the pharmacovigilance analysis rather than a causal relationship established in this study.

A key boundary of this study is that the pharmacovigilance and transcriptomic analyses were derived from different data contexts. The FAERS analysis evaluated drug-event reporting associations among adverse-event reports, whereas the scRNA-seq datasets were derived from sepsis patients and controls rather than ERA-exposed PAH patients who subsequently developed sepsis. Therefore, the Mono_IFN subset identified in the single-cell analysis cannot be interpreted as a mediator of ERA-associated sepsis reporting. Instead, Mono_IFN should be understood as a sepsis-associated interferon-responsive monocyte state that provides biological context for immune dysregulation in sepsis. The integration of these data layers was intended to generate hypotheses regarding potential overlap between PAH drug-target-related pathways and sepsis-associated immune programs, not to establish a causal pathway from ERA exposure to Mono_IFN expansion.

Importantly, the Mono_IFN program identified in this study should not be interpreted as specific to ERA exposure. IFITM3, ISG15, IFIT1, OAS1, and related interferon-stimulated genes are canonical components of antiviral and inflammatory responses and are frequently observed in viral infection, bacterial sepsis, and systemic inflammatory disorders. The current transcriptomic datasets do not contain drug-exposure-specific information for endothelin receptor antagonists, nor do they include monocytes experimentally treated with Maitentan, ambrisentan, or other ERAs. Therefore, our data cannot determine whether ERA exposure is capable of inducing an IFN-high monocyte state. The proposed connection between ERA-associated sepsis reporting and Mono_IFN-related immune dysregulation should be regarded as a biologically plausible but unvalidated hypothesis. Future studies using ERA-exposed PAH cohorts, prescription-linked transcriptomic datasets, or *in vitro* ERA-treated monocyte models will be required to test whether ERAs directly modulate interferon-responsive monocyte programs.

Third, the interaction between riociguat and IFITM3 is currently supported only by computational evidence derived from all-atom molecular dynamics simulations. The observed convergence of RMSD, stability of radius of gyration, reduction in solvent-accessible surface area, and persistence of hydrogen bond networks collectively indicate thermodynamic feasibility of the protein–ligand complex. However, a substantial experimental gap remains between computational prediction and functional validation. Direct binding affinity should be confirmed using biophysical approaches such as surface plasmon resonance (SPR) or isothermal titration calorimetry (ITC). Functional modulation of IFITM3 requires validation in cellular systems, including assessment of interferon pathway activity and cytokine release profiles. Ultimately, *in vivo* studies in sepsis models are necessary to evaluate the immunomodulatory and protective effects of riociguat. Until such evidence is available, the proposed repositioning of riociguat as a modulator of IFITM3 should be regarded as a testable mechanistic hypothesis rather than an established therapeutic strategy.

At the exploratory computational level, our molecular docking and MD analyses generated a riociguat–IFITM3 structural hypothesis. Riociguat is an approved soluble guanylate cyclase stimulator whose established pharmacological activity is mediated through the NO–sGC–cGMP pathway rather than through IFITM3. Therefore, the identification of riociguat in our docking screen should not be interpreted as evidence of IFITM3-targeted pharmacology.

In the present study, docking and 100-ns MD simulation suggested that riociguat could remain associated with a predicted IFITM3 structural model under the selected aqueous simulation conditions. However, these observations should be interpreted strictly as *in silico* evidence of structural compatibility. They do not establish direct biochemical binding, target engagement in cells, druggability of the predicted pocket, or functional modulation of IFITM3-mediated interferon or TLR-related signaling pathways. This limitation is particularly important because IFITM3 is a small membrane-associated protein, and AlphaFold-predicted structures of membrane-associated proteins require careful interpretation outside their native lipid environment. The present MD simulation did not include a lipid bilayer or membrane-context constraints and therefore cannot adequately recapitulate the native conformational or interaction landscape of IFITM3.

Accordingly, the riociguat–IFITM3 finding should be viewed only as a hypothesis-generating computational result. We do not propose riociguat as an IFITM3-targeted protective intervention, a mechanism-based treatment for sepsis, or a strategy to mitigate sepsis reporting associated with ERA therapy. Any potential biological relevance would require direct validation using biophysical binding assays, IFITM3-expressing cellular systems, monocyte-based interferon-response assays, cytokine readouts, and *in vivo* sepsis models. Clinical combination strategies involving PAH therapies cannot be inferred from docking or MD simulation and would require dedicated pharmacodynamic, safety, and outcome studies.

In summary, this study establishes a cross-scale analytical framework that integrates pharmacovigilance-derived safety signals with single-cell immune profiling and molecular-level modeling. Through this approach, PAH-targeted therapies—particularly endothelin receptor antagonists—are identified as a previously underrecognized class of drug exposures associated with sepsis risk. Mechanistically, we define the Mono_IFN subpopulation as a critical driver of immune dysregulation under conditions of interferon signaling overload, and develop a robust machine learning-based diagnostic model centered on IFITM3. Furthermore, molecular simulations suggest a potential strategy for targeting IFITM3 through riociguat, providing a conceptual basis for early immunomodulatory intervention. Collectively, this multi-scale paradigm—from population-level drug safety signals to cellular mechanisms and protein-level interactions—offers a systems biology blueprint for mitigating iatrogenic risk of severe infection in cardiopulmonary disease populations and warrants further experimental and clinical validation.

## Data Availability

Publicly available datasets were analyzed in this study. This data can be found here: All data used in this study are publicly available. The pharmacovigilance data were obtained from the FDA Adverse Event Reporting System (FAERS) database (https://www.fda.gov). Transcriptomic datasets were retrieved from the Gene Expression Omnibus (GEO) database under accession numbers GSE65682, GSE95233, GSE13904, GSE185263, GSE69528, and GSE63042. The single-cell RNA sequencing data analyzed in this study were obtained from publicly available datasets in GEO (accession numbers provided in the Methods section). Structural data for IFITM3 were obtained from the AlphaFold Protein Structure Database (https://alphafold.ebi.ac.uk). The three-dimensional structure of riociguat was downloaded from the PubChem database. All data processing and analysis were performed using publicly available tools and R packages as described in the Methods section. Custom scripts used in this study are available from the corresponding authors upon reasonable request.

## Supplementary material

SUPPLEMENTARY FIGURE S1 Original data cleaning and screening map of FAERS data. Adverse event reports related to septic conditions (HLGT = septic conditions) were initially extracted from the FAERS database (n = 142,039). After removing duplicate reports (n = 1,365), 140,674 cases remained. Reports were further restricted to those with the preferred term (PT) “Sepsis” (n = 96,024), and cases classified under specific sepsis-related conditions were excluded (n = 44,650). Subsequently, only reports in which drugs were identified as primary suspect (PS) or secondary suspect (SS) were retained (n = 68,061), while non-primary medication cases were excluded (n = 27,963). Cases without identifiable drug components (n = 9) and solvent-related records were further removed. After eliminating non-drug components and irrelevant entries (n = 1,318), a total of 66,734 sepsis cases were finally included in the analysis.

SUPPLEMENTARY FIGURE S2 WGCNA data preprocessing, construction of the weighted gene co-expression network, and functional enrichment analysis of key modules. **(A)** Boxplot of normalized gene expression levels across all samples in the GSE46955 dataset, verifying the consistency of data standardization prior to network construction. **(B)** Hierarchical clustering dendrogram of samples based on Euclidean distance to detect and eliminate potential outliers. **(C)** Analysis of network topology for soft-thresholding power selection. The left panel demonstrates the scale-free topology model fit i $ndex\left(R^{2}\right)asafunc$ tion of the soft-thresholding power, while the right panel illustrates the mean connectivity. A power of 16 was selected (indicated by the dashed line) to ensure a robust scale-free network. **(D,E)** Dot plots showing the top significantly enriched Gene Ontology (GO) Biological Processes **(D)** and Kyoto Encyclopedia of Genes and Genomes (KEGG) pathways **(E)** for the genes within the pink module. **(F,G)** Dot plots showing the top significantly enriched GO Biological Processes **(F)** and KEGG pathways **(G)** for the genes within the black module. In the dot plots **(D–G)**, the size of the dots represents the number of genes involved in the respective term (Count), and the color gradient indicates the adjusted P-value (p.adjust).

SUPPLEMENTARY FIGURE S3 Quality control assessment of multi-cohort gene expression data used for machine learning-based sepsis classification. **(A)** Sample size and class distribution across the six cohorts. Bars are colored by disease status: sepsis (red), healthy/control (blue), and SIRS (yellow, GSE63042 only). The training cohort GSE65682 exhibited the largest sample size n=802 with substantial class imbalance (sepsis:control = 760:42). **(B)** Class imbalance ratio (sepsis:control) for each cohort. The red dashed line indicates a balanced ratio of 1:1. Class-weighted approaches were applied during model training to account for the observed imbalance. **(C)** Distribution of normalized gene expression values after within-cohort Z-score standardization. (i) Boxplots and (ii) violin plots with embedded boxplots confirmed that all six cohorts were centered near zero with comparable interquartile ranges, validating the normalization procedure. Initial quality control identified that GSE69528 and GSE63042 had not undergone Z-score standardization in the original preprocessing pipeline; this was corrected prior to all downstream analyses, and post-correction distributions are shown here. **(D)** Missing value assessment across 33 consensus feature genes and six cohorts. All cells are green (zero missing values), confirming complete feature availability across all cohorts after intersection-based alignment. **(E)** Principal component analysis (PCA) of the integrated expression matrix colored by (i) cohort identity and (ii) disease status. PC1 explained 30.8% of total variance. GSE63042 showed partial separation from the remaining cohorts (annotated with arrow), attributable to its distinct sequencing platform (RNA-seq) and control group composition (SIRS rather than healthy individuals). The 95% confidence ellipses are shown for each group. Importantly, the remaining five cohorts demonstrated substantial overlap in PCA space, indicating successful cross-platform harmonization. **(F)** Uniform Manifold Approximation and Projection (UMAP) visualization colored by (i) cohort identity and (ii) disease status. Consistent with PCA findings, GSE63042 formed a partially distinguishable cluster (annotated), while the remaining cohorts showed considerable overlap. **(G)** Batch effect diagnostics. (i) Scree plot showing variance explained by each principal component (PC1–PC10). (ii) PC1 density plot stratified by cohort. The partial separation of GSE63042 along PC1 was driven by platform and control-type differences rather than systematic technical artifacts among the microarray-based cohorts, which displayed overlapping distributions. **(H)** Pearson correlation heatmap of the 33 consensus feature genes computed on the pooled expression matrix. Hierarchical clustering revealed two major co-expression modules: an interferon-stimulated gene cluster (IFIT1, IFIT3, OAS1, OAS2, MX1, ISG15, IRF7) and an antigen presentation/complement cluster HLA−DRA,HLA−DPA1,CD74,CTSS,C1QA,C1QB . The top annotation bar displays the mean expression difference between sepsis and control samples in the training cohort (GSE65682), with red bars indicating genes upregulated in sepsis and blue bars indicating genes downregulated in sepsis. **(I)** Density plots of the six most variable genes across all cohorts. Despite platform heterogeneity and the distinct control group in GSE63042, overall distribution shapes were broadly consistent, supporting the robustness of within-cohort Z-score normalization for cross-cohort feature comparability.

## Glossary

aOR: adjusted Odds Ratio
AlphaFold DB: AlphaFold Protein Structure Database;
ARDS: Acute Respiratory Distress Syndrome
AUC: Area Under the receiver operating characteristic Curve
AUCell: Area Under the Curve Cell
cDC: conventional Dendritic Cell
CGenFF: CHARMM General Force Field
CHARMM36: Chemistry at Harvard Macromolecular Mechanics 36 Force Field
CID: Compound Identifier
CI: Information Component
CellChatDB: CellChat Database;
cGMP: Cyclic Guanosine Monophosphate
DEGs: Differentially Expressed Genes
DTW: Dynamic Time Warping
ΔG: Gibbs Free Energy Change
ERA: Endothelin Receptor Antagonist
EPC: Edge Percolated Component
ET-1: Endothelin-1
ETA: Endothelin Type A Receptor
ETB: Endothelin Type B Receptor
FAERS: FDA Adverse Event Reporting System
FS: Feature Selection
GBD: Global Burden of Disease
GEO: Gene Expression Omnibus
GLM: Generalized Linear Model
GLMNET: Generalized Linear Model with Elastic Net Regularization
GO: Gene Ontology
GROMACS: Groningen Machine for Chemical Simulations
GSVA: Gene Set Variation Analysis
HLA: Human Leukocyte Antigen
HVGs: Highly Variable Genes
ICAM-1: Intercellular Adhesion Molecule 1
IFN: Interferon
IFITM3: Interferon-Induced Transmembrane Protein 3
IFNGR: Interferon Gamma Receptor
IL: Interleukin
IQR: Interquartile Range
ISGs: Interferon-Stimulated Genes
ITC: Isothermal Titration Calorimetry
JAK: Janus Kinase
KNN: k-Nearest Neighbors
LASSO: Least Absolute Shrinkage and Selection Operator
LDA: Linear Discriminant Analysis
LINCS: Linear Constraint Solver
MAD: Median Absolute Deviation
MCC: Maximal Clique Centrality
MD: Molecular Dynamics
MedDRA: Medical Dictionary for Regulatory Activities
MEs: Module Eigengenes
MCP-1: Monocyte Chemoattractant Protein-1
MHC: Major Histocompatibility Complex
MNC: Maximum Neighborhood Component
MMFF94: Merck Molecular Force Field 94
M- MDSCs: Monocytic Myeloid-Derived Suppressor Cells
NF-κB: Nuclear Factor kappa-B
NK: Natural Killer
NO: Nitric Oxide
NPT: Isobaric-Isothermal Ensemble
NVT: Canonical Ensemble
OR: Odds Ratio
PAH: Pulmonary Arterial Hypertension
PAGA: Partition-based Graph Abstraction
PCA: Principal Component Analysis
PDB: Protein Data Bank
PBMCs: Peripheral Blood Mononuclear Cells
PI3K: Phosphatidylinositol 3-Kinase
PME: Particle Mesh Ewald
PRR: Proportional Reporting Ratio; PS: Primary Suspect
PT: Preferred Term; Rg: Radius of Gyration
RF: Random Forest
ROC: Receiver Operating Characteristic
RMSD: Root Mean Square Deviation
RMSF: Root Mean Square Fluctuation
ROR: Reporting Odds Ratio
SASA: Solvent-Accessible Surface Area
scRNA-seq: single-cell RNA Sequencing
SHAP: SHapley Additive exPlanations
sGC: soluble Guanylate Cyclase
SPR: Surface Plasmon Resonance
SS: Secondary Suspect
STAT1: Signal Transducer and Activator of Transcription 1
SVM: Support Vector Machine
SVM-RFE: Support Vector Machine–Recursive Feature Elimination
ssGSEA: single-sample Gene Set Enrichment Analysis
TLR: Toll-Like Receptor
TIP3P: Transferable Intermolecular Potential 3 Point
TOM: Topological Overlap Matrix
TNF-α: Tumor Necrosis Factor-alpha
TTO: Time-to-Onset
UMAP: Uniform Manifold Approximation and Projection
WGCNA: Weighted Gene Co-expression Network Analysis
XGBoost: Extreme Gradient Boosting.
