# Supplementary figures and images for: Sepsis reporting signals associated with endothelin receptor antagonists and IFITM3-Centered interferon-responsive monocyte features: a pharmacovigilance and transcriptomic study
Source: Front Pharmacol. 2026 Jul 16;17:1864683. doi: 10.3389/fphar.2026.1864683 (PMC13421420; doi:10.3389/fphar.2026.1864683)

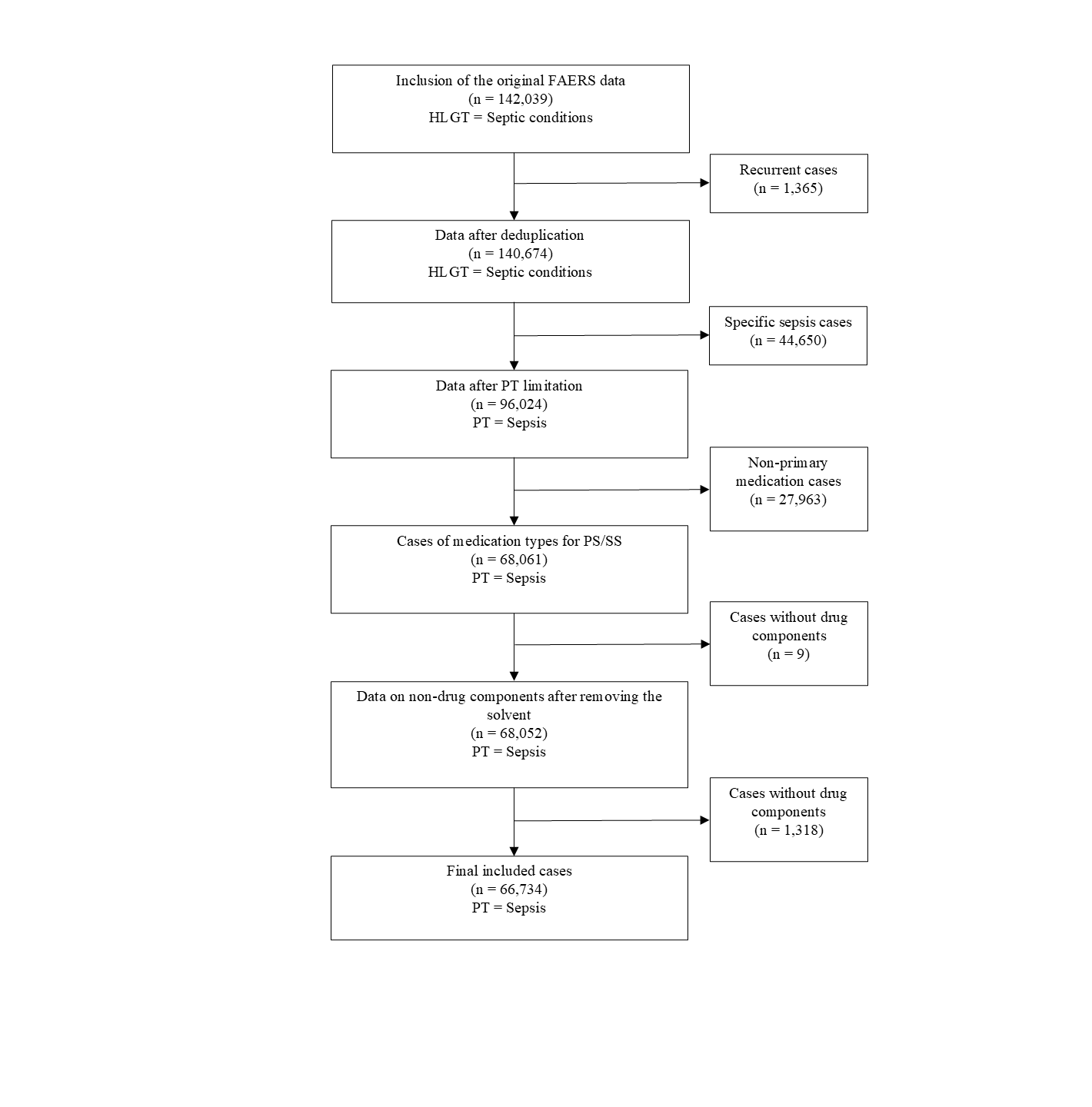

Supplement: Supplementary file 1 [file Image1.tiff]

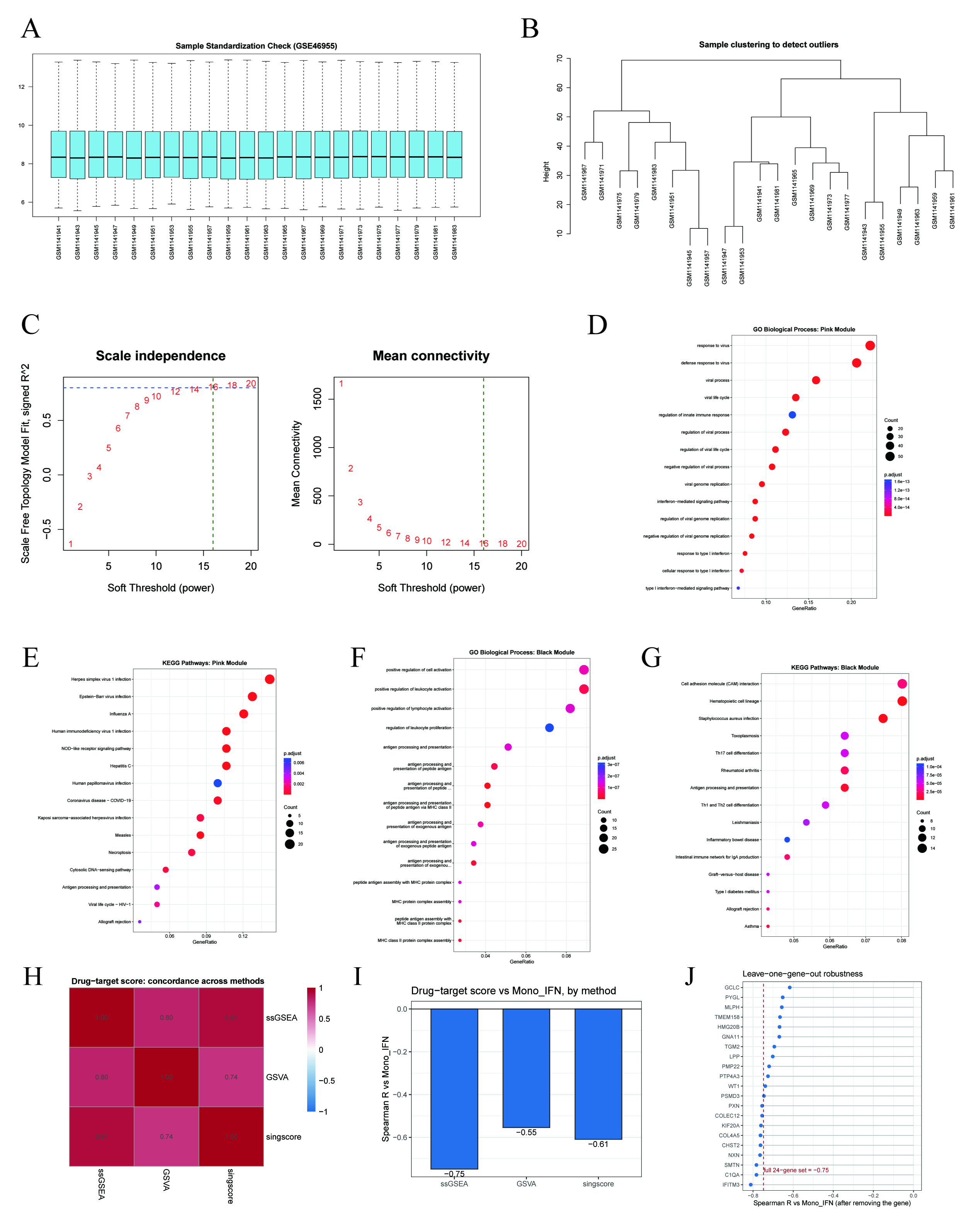

Supplement: Supplementary file 2 [file Image3.tif]

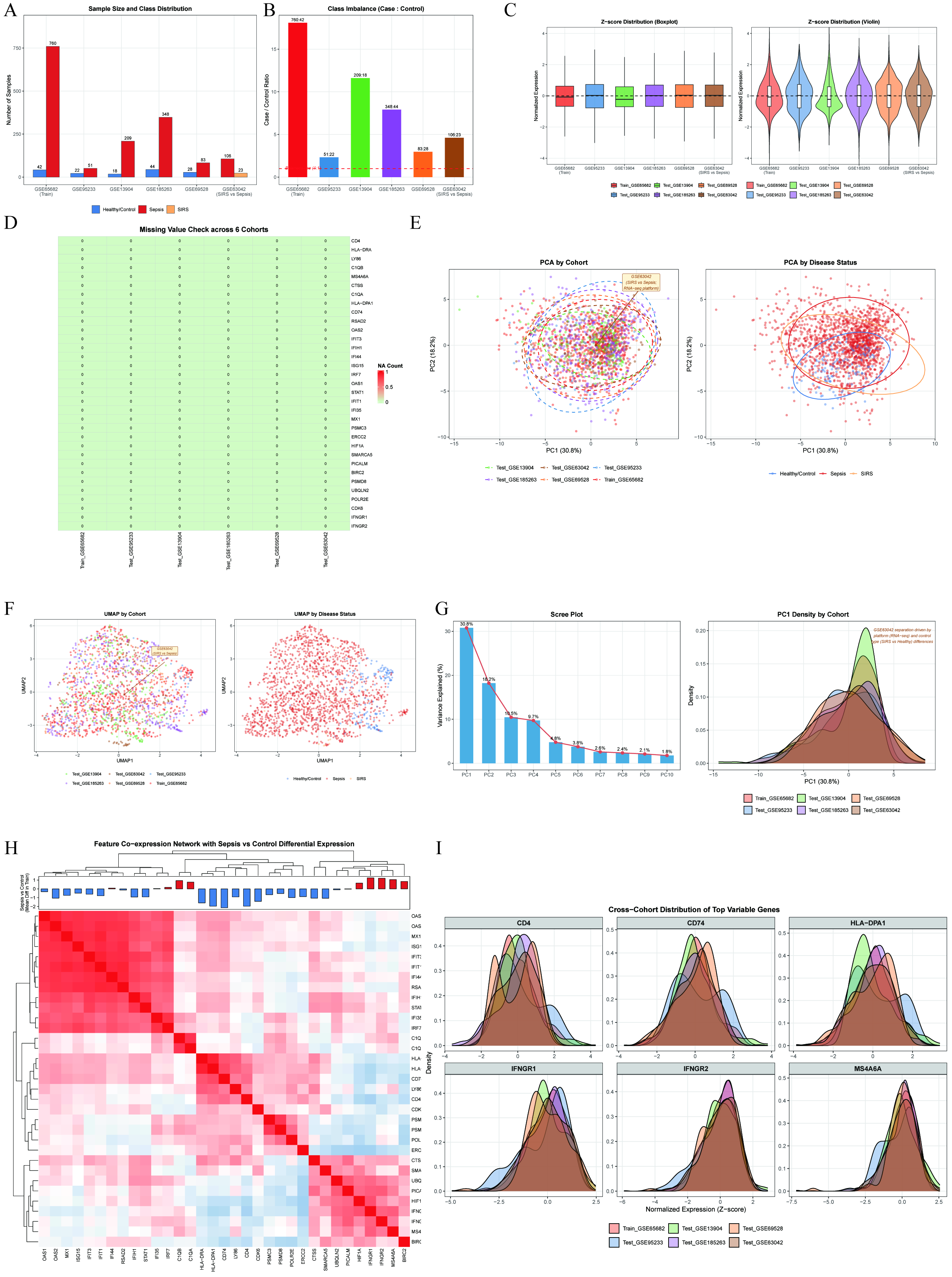

Supplement: Supplementary file 4 [file Image2.tif]
